# Potentially Toxic Elements in Soil of a Historical Mining Region in Serbia: Geochemical Fractionation, Ecological Impact, and Health Risk Assessment

**DOI:** 10.3390/toxics14070608

**Published:** 2026-07-12

**Authors:** Almasa Lekpek, Svetlana Đogo Mračević, Zoran Dinić, Aleksandra Šajnović, Slavica Ražić, Milan Stanković, Branimir Jovančićević

**Affiliations:** 1 Faculty of Chemistry, University of Belgrade, Studentski trg 12-16, 11000 Belgrade, Serbia; almasalekpek993@gmail.com (A.L.); bjovanci@chem.bg.ac.rs (B.J.); 2 Faculty of Pharmacy, University of Belgrade, Vojvode Stepe 450, 11221 Belgrade, Serbia; slavica.razic@pharmacy.bg.ac.rs (S.R.); milan.stankovic@pharmacy.bg.ac.rs (M.S.); 3 Institute of Soil Science, Teodora Drajzera 7, 11000 Belgrade, Serbia; soils.dinic@gmail.com; 4 Institute of Chemistry, Technology and Metallurgy, University of Belgrade, Njegoševa 12, 11000 Belgrade, Serbia; aleksandra.sajnovic@ihtm.bg.ac.rs

**Keywords:** chemical partitioning, mining legacy, environmental indices, soil contamination, human exposure

## Abstract

Natural metal enrichment, combined with centuries of mining activity in the Rudnik Mountain region of Serbia, has led to the long-term accumulation of potentially toxic elements (PTEs) in the soil. Although the investigated area lies outside the current zone of active exploitation, the persistence and mobility of accumulated PTEs, historical contamination can still affect the local population. This study integrated assessments of soil characterization, enrichment, sequential extraction-based fractionation, mobility, and bioavailability of As, Cd, Co, Cr, Cu, Fe, Mn, Ni, Pb, and Zn, as well as the associated ecological and human health risks. Geochemical and environmental pollution indices further identified As, Pb, and Cd as the main contributors to environmental risk. Sequential extraction results based on the BCR protocol indicated significant bioavailability of Cd (32% in the exchangeable fraction) and Pb (74% in the reducible fraction). In contrast, over 80% of As was present in the residual fraction, indicating limited mobility and low bioavailability. Health risk assessment for the local rural population revealed significant potential health concerns at the investigated sites, with exceedances of the hazard index (HI) and carcinogenic risk (CR) values, primarily associated with As as the main contributing pollutant.

## 1. Introduction

The exploitation of mineral deposits and the development of metallurgy throughout history have been a significant source of metal emissions into the environment [1,2,3,4]. Over the centuries, large quantities of mining and metallurgical waste—including tailings, slag, and smoke particles—enriched with potentially toxic elements (PTEs) specifically Pb, As, Cd, Cu, and Zn have been produced. These wastes remain long-term sources of pollution, as PTEs can be dispersed through the atmosphere and water, and pollution can persist for centuries or millennia after mining ceases.

Given the long-term persistence of mining-derived contaminants in the environment, identifying pollution sources in mining regions is essential for developing effective environmental management strategies and risk mitigation measures [3]. This is particularly important in historical mining districts, where numerous abandoned or poorly documented mining and smelting facilities no longer show visible signs of past activities yet may still be hotspots of PTE contamination. Consequently, soils near former mining operations often serve as long-term repositories of mining-derived pollutants and key indicators of environmental degradation.

Among PTEs, As, Cd, and Pb are widely recognized as persistent soil pollutants in areas affected by historical mining activities for decades to centuries after mine closure [3]. Their environmental impact is determined by total concentrations and interactions within the soil system [1,2,3,4]. Elements that are weakly bound to soil components can easily migrate through soil layers, be transported through drainage systems, or accumulate in alluvial deposits, increasing ecological and human health risks [4]. Therefore, a proper understanding of the speciation, mobility, and potential bioavailability of PTEs in these areas is essential for valid environmental monitoring and risk assessment. To better understand the long-term environmental behavior of PTEs, sequential extraction procedures are frequently used to determine the geochemical fractions in which these elements are retained within soils and mining residues [1,2,3,4].

Guillevic et al. [5] and Mariet et al. [5] showed that soils around historical French mining and smelting sites remain highly contaminated centuries after mining ceased. Pb persists as potentially mobile, primarily associated with Mn-Fe (hydr)oxides. Similarly, in historical Pb–Zn mining regions in Derbyshire, England, up to 50% of Pb was associated with the reducible fraction, whereas up to 52% of Cd occurred predominantly in the highly mobile exchangeable fraction [4]. Furthermore, a recent study of soils from decommissioned smelter sites in Brazil [2] reported that both Pb and Cd remained significantly associated with mobile soil fractions. Pb was predominantly associated with the reducible and oxidizable fractions, highlighting the important role of Fe–Mn oxides in controlling its mobility. Cadmium was predominantly extracted into the exchangeable and reducible fractions, suggesting significant active contamination, even 30 years after the smelter’s closure. Results emphasized that the long-term persistence of these elements is strongly influenced by total concentrations, mineralogical composition, secondary mineral formation, and landscape characteristics.

In this context, the historically active mining and metallurgical region of Mount Rudnik (Serbia) was selected as the study area. This is one of the oldest mining regions in the Balkans, characterized by a significant Pb-Zn-Cu-Ag polymetallic ore deposit [5]. Mining activities in the area have been continuous from approximately 2300 BC to the present, including the Roman and medieval periods [5]. As a result, substantial amounts of mining waste have been generated, making the area a critical site for investigating metal distribution.

With this understanding of geochemical history and the significance of the current elemental profile, this study focused on determining the concentrations of the PTEs As, Cd, Co, Cr, Cu, Fe, Mn, Ni, Pb, and Zn in soils from Rudnik Mountain. The research involved determining total metal contents and applying sequential extraction procedures to assess their distribution among geochemical fractions, as well as their mobility and potential bioavailability. Contamination risk assessment cannot rely solely on total metal content in soil; it also requires a detailed study of metal fractionation, as this governs complex physicochemical and biological processes, mobility, bioavailability, and ultimately the potential impact on the rural population. In this context, a comprehensive evaluation of PTE occurrence and behaviors is necessary to better understand their environmental and potential health implications. Additionally, an ecological risk assessment, supported by graphical analysis of risk indices, was carried out to evaluate the potential environmental impact. Finally, an assessment of non-carcinogenic and carcinogenic health risks for the local rural population was performed.

This study contributes to a better understanding of the long-term dynamics of PTEs resulting from historical mining activities and to a more reliable assessment of their impacts on the environment and human health, and to the development of a sustainable approach to this challenge.

## 2. Materials and Methods

### 2.1. Study Area

The region around Mount Rudnik is one of the most important metallogenetic areas in Serbia and southeastern Europe. The complex geological structure, combined with intensive magmatic, tectonic, and hydrothermal processes, has led to the formation of a significant Pb-Zn-Cu-Ag-Bi-W polymetallic sulfide ore deposit. The ore deposits are associated with hydrothermal skarn within the Oligocene-Miocene magmatic complex of the intrusive volcanic series [6,7,8]. The onset of Oligocene dacite and quartz latite volcanic activity has an absolute age of 31.9–30.0 Ma [9]. The size of the ore bodies usually ranges from 30,000 to 150,000 tonnes, with the largest reaching 300,000 to 900,000 tonnes. Key characteristics include complex sulfide-sulphosalt mineral associations dominated by pyrrhotite, with sphalerite, galena, and chalcopyrite as major ore minerals, accompanied by high metal contents (Pb 0.9–5.6 wt%, Zn 0.5–4.5 wt%, Cu 0.08–2.18 wt%, and Ag 50–297 µg/g) and ongoing mining of massive and veinlet ore types [10]. The mineralogical composition of the ore is highly complex, with more than 70 mineral species identified, including native bismuth, bismuthinite, cosalite, galenobismuthite, vikingite, scheelite, and several Ni sulfides and arsenides [8]. Today, this area is of great economic importance due to the extraction of sulfide deposits of Pb, Zn, and Cu, with Ag present as an associated component. Extraction is carried out primarily through underground mining, with approximately 240,000—290,000 tonnes of ore processed annually [11].

Although today’s active mines are located on the southwestern slopes of Rudnik Mountain, the entire region has a rich mining history. Mining dates back to 2300 BC and is associated with the Bubanj Hum III culture and the extraction of Cu ores [5]. During the Roman period, Rudnik was one of the most important mining and metallurgical centers in the central Balkans, where Ag, Pb, Cu, and Fe ores were extracted. In the Serbian medieval state, Rudnik was also a major center for the extraction and processing of Ag, Pb, and Fe ores [12,13]. Numerous ore processing and smelting sites were located throughout the mountain and the surrounding forest areas. Centuries of ore exploitation and processing may have contributed to the accumulation of potentially toxic elements in the soil, not only in the area of the old mines, but also in the tailings and transport routes. Therefore, current soil contamination at certain locations may be linked not only to modern mining activities but also to historical mining and smelting processes throughout the mountain.

**Figure 1:**
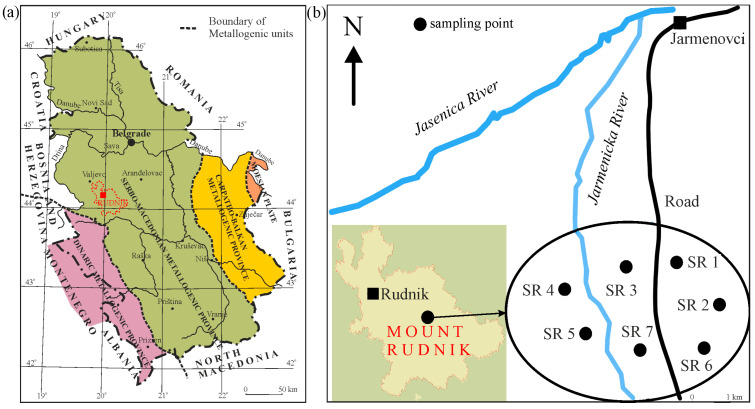
Study area and location of sampling sites. ((**a**) is modified and adapted from the Geological Atlas of Serbia [15] [In Serbian]; (**b**) is originally created by the authors).

The study area (Figure 1) is located in central Serbia, at approximately 44° north latitude and 20° east longitude, on the slopes of Mount Rudnik, between the municipalities of Gornji Milanovac and Topola, in the upper reaches of the Jarmenička River, about 80 km south of Belgrade. The terrain is mountainous, with the highest peak (Cvijićev vrh) reaching approximately 1132 m above sea level. Forests are dominated by beech and oak at lower elevations, while higher elevations are characterized by pastures. The climate is moderate continental, with average annual temperatures between 8 °C and 11 °C. Annual precipitation is relatively evenly distributed, with peaks in spring and autumn, and typically ranges from about 700 mm [14]. Snow is common in winter. Households and arable land are situated on the lower mountain slopes downstream from the sampling site, where local residents and tourists often engage in recreational activities including resting, gathering wild edible plants, and collecting firewood.

### 2.2. Sampling, Sample Preparation, and Extraction

Sampling was carried out in spring 2025, following a 10-day period without significant precipitation, to minimize variability from short-term weather conditions. The sampling sites were selected using a hydrology- and risk-oriented transect approach. Sites were strategically positioned in the middle reach of the river system, upslope and upstream of densely populated residential areas. This criterion was adopted to evaluate the downstream transport of PTEs along the river corridor and to assess immediate environmental risks to human receptors located further downstream within the catchment.

All samples were collected according to the standard procedure [16], using a sampling probe from a depth of 30 cm in the surface soil at seven sampling points. At each site, five subsamples were collected and combined to form a composite homogeneous sample in accordance with the LUCAS 2018—SOIL COMPONENT protocol [17]. Samples were placed in polyethylene bags and transported to the laboratory. External materials, including rhizomes and animal residues, were carefully removed. To prevent contamination and degradation, soil samples were air-dried for 1 week under well-ventilated, dark conditions, then homogenized and sieved (<2 mm) prior to further analysis.

Particle-size distribution was determined using the pipette method [18]. Textural classes were determined according to the International Union of Soil Sciences soil texture classification system using the soil texture triangle [19]. Soil types were classified according to the IUSS Working Group WRB system for soil classification and nomenclature [20]. The classification followed the World Reference Base for Soil Resources (WRB) criteria [21]. The pH in H_2_O and 1 M KCl was determined using the potentiometric method [22]; CaCO_3_ (%) by the volumetric method [23]; and organic matter (OM) content according to SRPS ISO 10694:2005 [24].

Soil samples were processed and analyzed using harmonized, standardized protocols suitable for environmental contamination studies: pseudototal PTE concentrations were quantified after aqua regia digestion in accordance with the International Organization for Standardization standard ISO 54321:2020. After digestion, the cooled samples were filtered through a 0.45 µm membrane filter and diluted with deionized water to a final volume of 50 mL.

To further characterize metal fractionation and potential mobility, the modified BCR (Community Bureau of Reference) sequential extraction procedure was applied [25]. This method fractionates elements into four operationally defined fractions: exchangeable/acid-soluble (F1), reducible (F2), oxidizable (F3), and residual (F4), representing progressively less mobile and bioavailable forms. Extraction was performed according to the standardized BCR protocol. The resulting fractions were used to evaluate metal mobility, bioavailability, and environmental risk. Analytical blank solutions were prepared using the same procedure as the reagent quality control. All analytical results for PTE concentrations are calculated and expressed on an oven-dried (dry weight) basis.

### 2.3. Instrumental Analysis and Method Validation

An inductively coupled plasma optical emission spectrometer with an axial view Thermo Scientific iCAP 6300 Duo ICP-OES (Thermo Fisher Scientific, Waltham, MA, USA) was used to determine the concentration of PTEs [26]. Quantification was performed using external calibration curves tailored to the expected elemental concentrations. For elements at low trace levels (Cd and Co), a basic calibration was performed using five standard solutions over the range of 0.01–2.0 mg/L. For the remaining elements (As, Cr, Cu, Mn, Ni, Pb, and Zn), calibration standards ranged from 0 to 10 mg/L, while the calibration range for Fe was extended to 0–25 mg/L. The resulting correlation coefficients for all calibration curves were greater than 0.99. To ensure that elements with higher concentrations (Fe, Pb, Mn, and Zn) fell within the linear calibration range, the sample solutions, initially diluted approximately 33-fold for aqua regia digestion and 40-fold for sequential extraction, were further diluted 100-fold prior to measurement.

High-purity argon (≥99.999%) was used for both plasma generation and purging the optical system. The optimized operating conditions were as follows: radio frequency (RF) of 27.12 MHz, plasma power of 1.15 kW, peristaltic pump rate of 50 rpm, and nebulizer gas flow of 0.5 L/min.

Elemental analysis was performed at the following emission wavelengths (nm): As 189.0, Cd 214.4, Co 228.6, Cr 267.7, Cu 327.3, Fe 259.9, Mn 260.5, Ni 231.6, Pb 220.3, and Zn 213.8.

The limits of detection (LODs) and limits of quantification (LOQs) were calculated as three and ten times the standard deviation of ten independent blank measurements, respectively (Table S1). Method accuracy was evaluated using recovery tests with the certified reference materials Loam Soil (ERM^®^-CC141) and EUROSOIL 7 (IRMM-443-7). Recovery rates ranged from 88% to 111%, confirming acceptable method performance.

Validation of the BCR extraction procedure was carried out using recovery percentages calculated as the ratio of the sum of the F1-F4 fractions to the pseudo-total PTE concentrations [27]. The recovery percentages ranged from 87.9% to 117.6% (Table S2).

The statistical analysis was performed using Microsoft Excel 365 (Microsoft, Redmond, WA, USA). Spearman rank correlation coefficients were used to evaluate trends in both total concentrations and BCR fractions. Censored data below the limit of detection (<LOD) in the carbonate fractions were replaced with half of the detection limit (LOD/2) prior to statistical evaluation. Censored data below the limit of detection (<LOD) observed in both the carbonate content and Cr content in the exchangeable BCR fraction were replaced with half of their respective detection limit (LOD/2) prior to statistical evaluation and correlation analyses.

### 2.4. Geo-Accumulation and Ecological Risk Assessment

The concentrations of potentially toxic elements (PTE) in the tested soil samples were compared with the corrected maximum allowable concentrations (CMAC) and the corrected remediation concentrations (CRC) in accordance with the Regulation on Limit Values of Polluting, Harmful and Hazardous Substances in Soil of the Republic of Serbia. The comparison took into account the clay and organic matter content, which affect metal retention and mobility in soil (Table S3) [28].

Due to the lack of pre-industrial data on metal concentrations in the surface soil layer in Serbia, the environmental impact of PTE contamination was assessed using the composition of the upper continental crust (UCC) as a geochemical reference [29,30]. To evaluate the relative enrichment of soils compared to the average UCC composition, concentrations of potentially toxic elements (PTEs) were normalized to UCC values. In addition, the enrichment factor (EF) and the geo-accumulation index (Igeo) were calculated (Table S4). The enrichment factor (EF) is commonly used as a quantitative measure of soil pollution, specifically to identify anthropogenic enrichment [29,31]. To better understand PTE enrichment levels in soil and assess contamination from extreme accumulation, the geo-accumulation index (Igeo) was used [32]. Furthermore, to assess the contamination status of individual sampling sites, the pollution load index (PLI) and the Nemer index (NI) were applied. The PLI, based on average soil quality, provides a general indication of contamination intensity, while the NI emphasizes the impact of elements with extreme enrichment [32].

To assess the ecological risk associated with PTE, the Ecological Risk index (ERI), Potential Ecological Risk Index (PERI), Risk Assessment Code (RAC), and Bioconcentration Factor (BF) were applied [33,34,35]. The ERI assesses the ecological risk of individual elements, considering both their concentrations and toxic response factors, while the PERI provides an integrated assessment of the overall ecological risk at each sampling site [32,36,37,38]. All equations, classification criteria, and results are presented in the Supplementary Materials (Tables S4–S8). In soils with high enrichment but variable PTE bioavailability, the Risk Assessment Code (RAC) provides important information for environmental risk evaluation because it considers not only the total concentrations of elements but also their mobility and bioavailability, thereby reflecting the immediate environmental risk associated with the most mobile and readily bioavailable forms of PTEs [39,40]. Furthermore, because elements associated with the reducible fraction can also be mobilized under variable environmental conditions, the Bioavailability Factor (BF), defined as the ratio of the sum of the first two sequential phases to the sum of all four phases, was also calculated [35]. Therefore, while RAC assesses current mobility and immediate environmental risk, BF provides information on the potential mobility and future bioavailability of PTE [41,42]. In addition, to assess which elements pose the greatest environmental threat, the potentially mobile fraction (PMF), defined as the sum of element concentrations in F1–F3 relative to the total, was calculated (Table S9) [33,35]. The combined application of RAC, BF, PMF, ERI, and PERI enabled a more comprehensive assessment of the environmental risk associated with PTE concentrations, mobility, bioavailability, and ecological factors in the studied soils [40].

### 2.5. Assessment of the Health Risk

**Figure 2:**
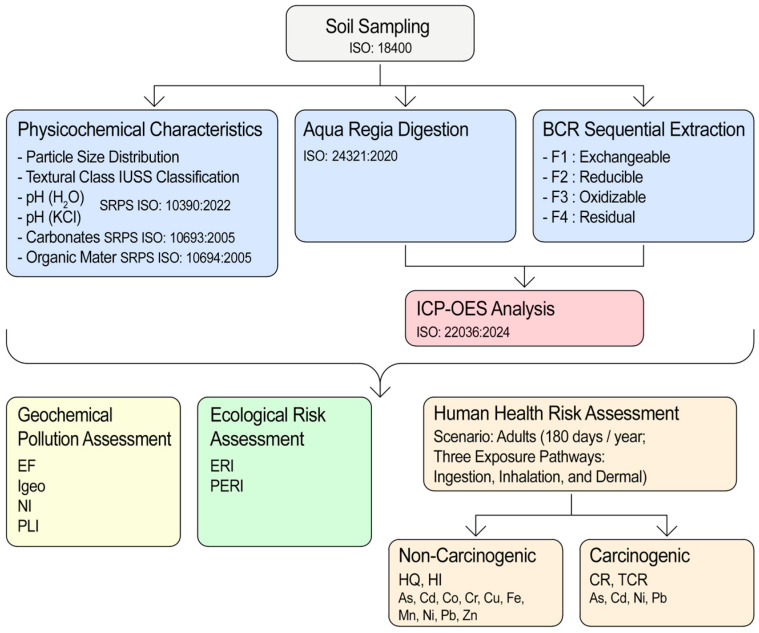
Flowchart of the experimental design.

To assess the risk to human health associated with the examined PTEs, the human health risk model recommended by the United States Environmental Protection Agency (USEPA) was applied (Tables S10–S12) [43]. As the sampling area is located in the hills above the populated zone, a seasonal exposure period of 180 days per year was used for the local rural population. This reflects the active period of approximately six months, during which the local population visits the area to walk or collect wild plants, mushrooms, and firewood. The risk was evaluated for both non-carcinogenic and carcinogenic effects across three exposure routes: ingestion, dermal contact, and inhalation. All parameters, equations, and risk assessment criteria used are presented in the Supplementary Material. The non-carcinogenic risk was assessed using the Hazard Quotient (HQ) for individual elements, while the Hazard Index (HI) was calculated for all sampling locations. The carcinogenic risk from exposure to As in soil was assessed using a recently updated SF of 32 mg/kg day, derived from the latest epidemiological studies on lung and bladder cancer and indexed in the US EPA IRIS database in 2025 [44,45]. For Cd and Ni, carcinogenic risk was evaluated only for the inhalation pathway as these elements are classified by the USEPA as probable human carcinogens primarily via inhalation, and no oral cancer slope factor (SF) has been established. Inhalation carcinogenic risks have been identified only for Ni species associated with occupational and industrial exposures, including Ni subsulfide and refinery dust [46,47,48]. Because there is no safe lower intake limit for Pb, the US EPA does not have an official SF for this metal. For comparison with the scientific literature, the substitution factor of 0.0085 mg/kg-day recommended by the California Environmental Protection Agency (CaIEPA) was used [49]. Cumulative cancer risk was calculated as the sum of cancer risks across all estimated PTEs and exposure pathways. The flowchart of the experimental design is presented in Figure 2.

## 3. Results and Discussion

### 3.1. PTE Distribution

The granulometric composition parameters of the investigated soil samples are presented in Table S13. The results show that particles larger than 2 mm range from 43% to 58% across the samples, with an average value of 51.5%, indicating a strongly skeletal soil. The fine fraction (<2 mm) ranges from 41% to 57%, with an average of 48.5%. All analyzed samples were classified as clay loam according to the IUSS soil texture triangle. In terms of soil type classification, samples SR3 and SR7 were identified as Fluvisols, while all other samples were classified as Leptosols according to the WRB classification system of the International Union of Soil Sciences.

The descriptive statistics for the ten investigated PTEs and the physicochemical characteristics of the soil samples are presented in Table 1 and Table S14. The observed difference between pH (H_2_O) and pH (KCl) indicates a high potential acidity, suggesting a strong potential for PTE mobilization in the soil with minor environmental changes. Soil samples collected near the river (SR3 and SR7) are less acidic, with carbonate contents of 2.96% and 0.09%, respectively. Carbonate content in all other samples was below the detection limit of 0.04%. Although the data set is limited in carbonate measurements, the results suggest a strong relationship with increased pH, indicating the important role of carbonates in controlling soil acidity. Soil organic matter averaged 9.1%, ranging from 6.51% to 15.1%. The average clay content was 20.9% and did not vary significantly between samples. The median concentrations of the ten investigated PTEs are as follows (Table 1): Fe (48,378 mg/kg) > Mn (1339 mg/kg) > Pb (1151 mg/kg) > Zn (314.52 mg/kg) > Ni (105.91 mg/kg) > As (81.8 mg/kg) > Cr (69.53 mg/kg) > Cu (51.03 mg/kg) > Co (21.94 mg/kg) > Cd (1.87 mg/kg). Except for Fe and Mn (which have no defined MAC values) and Cr, the median concentrations of all investigated elements exceed the maximum allowable concentrations set by Serbian and WHO standards. Moreover, the observed median concentrations of As and Pb exceed the remediation values established by Serbian standards for these elements [28].

For most of the analyzed PTEs, the standard deviation exceeded 20%, indicating significant spatial heterogeneity and uneven element distribution across the sampling sites. To assess average variability among samples, the coefficient of variation (CV) was used [35,50]. The CV values indicate that pronounced heterogeneity and high variability are often associated with localized enrichment zones. According to the classification criteria [50,51], Co and Fe with low variability (CV < 20%) and Ni, Mn, Cu, and Cr, with moderate variability (20–50%) have relatively uniform spatial distributions, suggesting they are mainly derived from the parent material. The As, Cd, and Zn in the high-variability category (50–100%) and Pb, with a CV over 100% (extreme-variability category), are attributed to anthropogenic activities. The CV is a measure of relative dispersion and does not provide direct information about the source of variability [52]. Therefore, the results may not indicate anthropogenic pollution.

**Figure 3:**
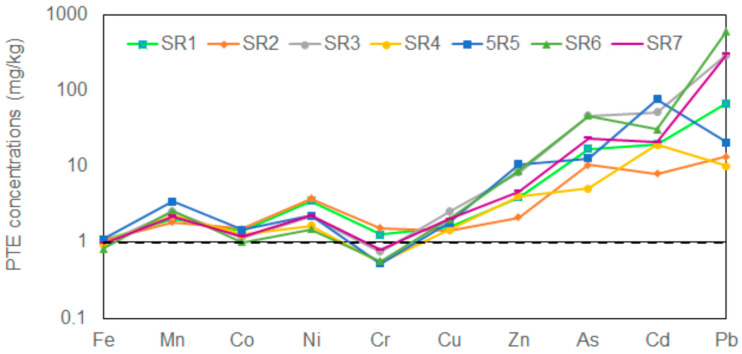
Diagram of PTE concentrations normalized to the upper continental crust.

To evaluate the distribution and enrichment of PTEs relative to the Upper Continental Crust (UCC), a UCC-normalized multielement diagram was generated directly from measured concentrations. The results (Figure 3) showed a similar distribution of PTEs across all analyzed samples, indicating common sources and similar geochemical processes throughout the study area. Based on these results, the elements can be grouped into three categories: (I) Fe, Mn, Co, Cr, and Ni; (II) Zn and Cu; and (III) As, Cd, and Pb.

Concentrations of elements in the first group across all samples cluster around the reference line, indicating a predominantly natural (geogenic) origin. The Zn and Cu show a slight upward trend, reflecting the soil’s specific mineral composition, providing an initial indication of anthropogenic activity. The highest enrichments are observed for As, Pb, and Cd, whose values significantly deviate from the average content in the Earth’s surface layer. In samples SR6, SR3, and SR7, Pb concentrations are several hundred times higher than the UCC values, clearly indicating a strong anthropogenic influence. Furthermore, the spatial distribution of PTEs across the study area appears to be strongly dependent on local hydrological processes and landscape characteristics. Sites SR3 and SR7 are located on alluvial soils developed on the floodplains of the Jarmenička River. PTE enrichment at these two sites is primarily driven by fluvial transport and subsequent deposition of contaminated upstream sediments during peak water discharge periods. Site SR6 is positioned at a higher elevation directly above SR7; consequently, the high PTE concentrations at the low-lying SR7 site are further enhanced by gravity-driven surface runoff and lateral sediment transport from SR6. The high Pb concentration detected at SR1 may be attributed to the site’s geomorphological characteristics. Situated on relatively flat terrain, the site functions as a local depositional area for materials transported from surrounding higher elevations, resulting in the gradual accumulation of lead in the soil.

### 3.2. Assessment of PTE Contamination

**Figure 4:**
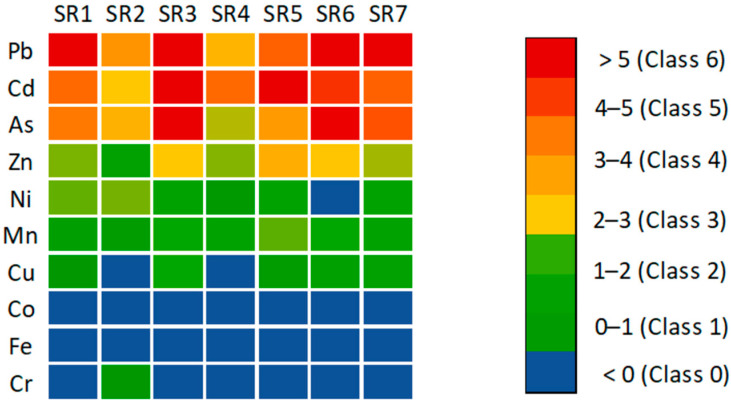
Distribution of the analyzed PTEs across different Geoaccumulation Index (Igeo) classes.

To further distinguish natural lithogenic variations from anthropogenic influences on PTE concentrations, enrichment factors (EFs) and geoaccumulation indices (Igeo) were calculated (Tables S15 and S16). The enrichment factor is commonly used to assess the relative contributions of natural and anthropogenic sources of PTEs in soil. EF values below 2 indicate a predominantly geogenic origin, whereas values above 5 indicate a significant anthropogenic contribution [53]. For the EF calculation, Fe was selected as the reference element because its measured concentration in the samples is close to the UCC value, indicating a natural, geogenic origin with minimal environmental impact. Based on EF values, elements can be classified into three groups: (I) Cu, Co, Mn, and Ni, with average EF values of 1.89, 1.28, 2.46, and 1.44, respectively, are considered predominantly of natural origin; (II) Zn and Cr, with EF values of 6.29 and 8.65, respectively, indicate a strong anthropogenic impact; and (III) As, Cd, and Pb, with extremely high EF values, indicating serious contamination and a dominant anthropogenic origin. To further assess soil contamination by PTEs, particularly those showing significant enrichment, the Igeo coefficients were calculated. Compared to the enrichment factor, Igeo provides a more robust measure of contamination intensity [38]. The average Igeo coefficients decrease in the order: Pb > Cd > As > Zn > Mn > Ni > Cu > Co > Fe > Cr. Based on the Igeo classification (Figure 4, Table S4), the examined soil samples are categorized as uncontaminated for Fe, Co, Cr, Cu, Mn, and Ni (Class 0 and Class 1); moderately contaminated for Zn (Class 2); and extremely contaminated for As, Cd, and Pb (Class 5 and Class 6). To assess the cumulative impact of individual PTE indices and provide a comprehensive evaluation of soil degradation, the Pollution Load Index (PLI), the Nemerow Pollution Index (NI), and the Potential Ecological Risk Index (PERI) were used (Table S6). The PLI values indicate widespread accumulation of multiple elements: site SR4 (PLI = 2.60) is classified as moderately contaminated, while all other samples with PLI values above 3.00 are classified as severely contaminated [54]. This cumulative impact is further highlighted by NI values exceeding 3.00 across all analyzed samples (ranging from 3.34 (SR2) to 8.85 (SR6), indicating extremely high pollution at all investigated sites.

While the PLI indicates total metal accumulation, the NI is more sensitive to extreme enrichment of specific elements [55]. Agreement between the evaluation categories of the two indices confirms the reliability of the assessment and shows that the pollution results are not driven by the large impact of a single element but by the accumulation of multiple anthropogenic elements.

### 3.3. Evaluation of Ecological Risk

**Figure 5:**
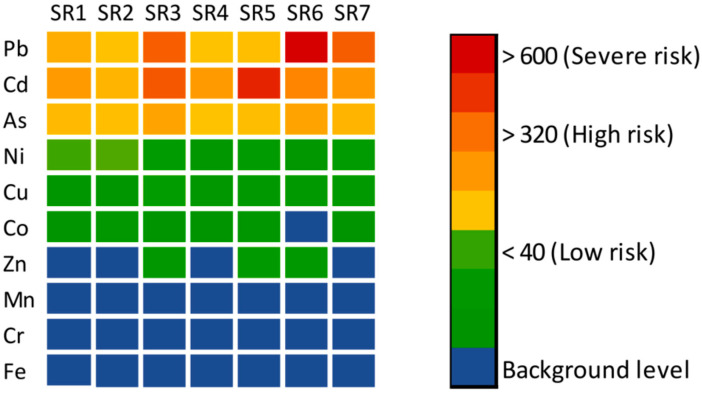
Distribution of the analyzed PTEs across different Ecological Risk Index (ERI) categories.

To assess soil pollution, it is insufficient to consider only the soil PTE load; it is also necessary to evaluate the potential implications for the surrounding biota. To evaluate ecological risk, this study adopted the Ecological Risk Index (ERI) and the Potential Ecological Risk Index (PERI) (Tables S6 and S7). Unlike geochemical indices, ecological indices incorporate a toxic response factor that varies according to the threat each element poses to the environment [35,56,57]. Elements with high toxicity, specifically Cd, Hg, As, and Pb, contribute more to the PERI even when their concentrations are significantly lower than those of less toxic elements [58]. As shown in Table S6 and Figure 5, the ecological indices for most of the studied PTEs are below 40, indicating low environmental risk. The As, with an average ERI of 231, falls into the high-risk category. In contrast, Pb and Cd are classified as very high risk, with ERI values exceeding 320. At certain sites (SR3 and SR5 for Cd, and SR3, SR6, and SR7 for Pb), these values reach severe ecological risk levels, with EF exceeding 1200. Furthermore, PERI values exceeded 600 in all investigated samples, indicating an extreme ecological risk throughout the area. In summary, combining geological indices with ecological risk assessments indicates that Pb, As, and Cd present considerable environmental risk.

To further support the findings and identify interactions between measured soil parameters and potential emission sources, Spearman correlation analyses were conducted for PTE content, soil pH, OM, and clay (Table S21). Given the limited sample size (n = 7), correlations were classified as weak (r_s_ < 0.50), moderate (0.50 < r_s_ < 0.75), or strong (r_s_ ≥ 0.75), where values exceeding 0.75 and 0.88 represent statistically significant (*p* < 0.05) and highly statistically significant (*p* < 0.01) correlations, respectively [59]. Strong Spearman coefficients were also found for the PTE pairs Pb-As (r_s_ = 0.89), Mn-Cd (r_s_ = 0.86), Cd-Zn (r_s_ = 0.93), Cr-Ni (r_s_ = 0.75), and Mn-Zn (r_s_ = 0.96). In contrast, strong negative correlations were found between clay and As (r_s_ = −0.76); Mn and Cr (r_s_ = −0.89) and Zn (r_s_ = −0.96); and Co and As (r_s_ = −0.75). The strong positive correlations among elements suggest a common origin, indicating that they may be derived from shared or closely related emission sources. Moderate correlations likely reflect partial overlap of sources and similar geochemical behavior in the environment. In contrast, negative correlations between clay and As indicate that As distribution and transport are not significantly related to adsorption processes on clay minerals.

### 3.4. Evaluation of PTE Bioavailability Based on Sequential Extraction

**Figure 6:**
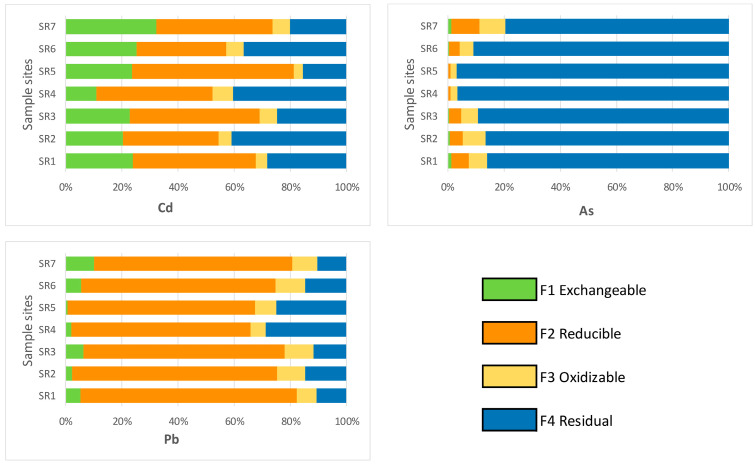
Distribution of As, Cd, and Pb in soil fractions based on the BCR sequential extraction procedure.

Although total element concentrations, their correlations, and geochemical and ecological risk parameters provide a general overview of environmental behavior, they do not reflect the actual bioavailability of PTEs. To address this, the BCR procedure was conducted to assess the chemical fractionation and mobility of the elements [60]. Results are presented in Figure 6 and Tables S17–S20.

Analysis of PTE distribution across BCR fractions provides important insights into mobility, stability, and environmental risks. Fractions F1–F3 are considered potentially mobile under changing environmental conditions, whereas F4 is immobile. To assess which elements pose the greatest environmental threat, the potentially mobile fraction (PMF) was calculated [61]. The average PMFs of the investigated elements decrease in the following order: Pb (89%) > Mn (83.8%) > Cd (75.9%) > Co (45.4%) > Zn (23.6%) > As (17.3%) > Cu (14.4%) > Ni (8.6%) > Cr (6.9%) > Fe (5.8%). Based on these results, three main groups can be identified: (I) low mobility, with PMF below 20% (As, Cu, Ni, Cr, and Fe); (II) moderate mobility, with PMF between 50% and 20% (Co and Zn); and (III) high mobility, with PMF above 50% (Pb, Mn, and Cd).

The group of elements with low mobility is dominated by Fe and has the smallest share in the PMF (3.30–7.36%). This suggests that Fe is highly stable in soil, likely originating from primary minerals including hematite and magnetite, as well as Fe species in clay minerals [60,62,63]. In this form, Fe is poorly mobile and has a reduced capacity to bind other elements due to its limited surface area and reactivity [64]. Because Fe (oxy)hydroxides, particularly in their hydrated, reactive forms, play a crucial role in immobilizing PTEs in soils, this could be an adverse effect, limiting the mobility and bioavailability of these elements [65]. Cr, Cu, Ni, and As exhibit weak mobility across all examined samples, indicating stabilization in the soil, likely through incorporation into the silicate matrix or Fe oxides [64].

Despite high concentrations in all tested samples, increased enrichment factors, and environmental risk assessment, the results of As distribution across sequential extraction fractions suggest a different interpretation of its environmental effects. The predominance of As in the residual fraction indicates that it is incorporated into a stable mineral matrix, is environmentally stable, and has very limited bioavailability. Given its toxicity, this element will still be included in further environmental and human health assessments.

The total potential mobility of Zn is below 20% in most analyzed samples (SR1, SR2, SR4, SR5, and SR6), indicating that it is predominantly associated with the stable residual fraction and exhibits low mobility in the system. Two samples in direct contact with water (SR3 and SR7) show slightly higher PMF values of 25.4% and 28.1%, respectively, suggesting increased mobility, likely due to different rare interaction conditions with water. The Zn in the residual fraction could be associated with poorly soluble minerals, specifically hematite, goethite, and hemimorphite [66]. In contrast, some studies [66,67,68] reported a significant portion of Zn in the oxidizable fraction, suggesting that organic matter is a natural sink for this element in soil.

A considerable portion of Co (45%) is present in potentially mobile fractions, predominantly in the reducible fraction (40% of the total content), suggesting an association with Mn and Fe (oxyhydr)oxides. These findings are consistent with previous studies [60].

Manganese’s high contribution to the potentially mobile fraction was expected, as it typically occurs in soils as chlorides and sulfates (in the exchangeable fraction), oxides, carbonates, and Mg-Fe oxides (in the reducible fraction), and as sulfides, usually associated with organic matter [60,69,70]. In the reducible fraction, Mn occurs as silicates and aluminosilicates [60]. The Mn in the reducible fraction plays a crucial role in the temporary immobilization of PTEs through coprecipitation and adsorption [71].

The distribution of Cd across the BCR fractions was as follows: reducible (34–58%) > residual (15–40%) > exchangeable (11–32%)> oxidizable (4–7%), indicating a heterogeneous distribution among the samples. The proportion of Cd in the residual fraction suggests that a moderately significant amount is stably incorporated within the mineral matrices. Cd is classified as an extremely toxic element with high mobility [60], and its predominance in the bioavailable fractions raises potential environmental concerns. Cd in the bioavailable fractions is primarily associated with chlorides, oxides, and hydroxides [72]. Additionally, due to its lower electronegativity and larger hydrated radius, Cd has a weaker binding affinity for Fe (oxyhydr)oxides than Pb, Cu, and Zn [72], which may contribute to its high mobility.

The distribution of Pb across sequential extraction phases was as follows: 0.58% (SR5) to 10% (SR7) in the exchangeable fraction, 64% (SR4) to 77% (SR1) in the reducible fraction, 5% (SR4) to 10% (SR3 and SR6) in the oxidizable fraction, and 10% (SR7) to 28% (SR4) in the residual fraction. The Pb is an element whose severe environmental and human health effects depend strongly on its speciation in soil [72]. The Pb content in the residual fraction indicates that a moderately high percentage of this metal is incorporated into a stable mineral matrix. Previous studies [60,62,69,73] indicate that the Pb exchangeable fraction contains mainly soluble Pb halides, and the reducible fraction contains less soluble Pb species including sulfates, carbonates, and chromates. The low Pb content in the oxidizable fraction suggests that organic substrates are not the primary sink for this metal. The Pb occurs naturally most often as galena ore (PbS), and, depending on its grain size, it can be extracted as the oxidizable or residual fraction [60].

To identify the key factors that statistically significantly influence the fate of PTEs in the environment and to assess their affinity for different soil fractions, a correlation analysis was conducted between the PTE content of each BCR phase and soil pH, clay content, and organic matter. Due to the limited number of samples (n = 7), the pronounced spatial heterogeneity of the matrix, and potential non-linear relationships, Spearman’s rank correlation analysis was performed (Tables S22–S25). The interpretation followed the same strict evaluation criteria established previously where (r_s_ ≥ 0.75) indicates a strong correlation and statistical significance (*p* < 0.05) and correlations with (r_s_ ≥ 0.88) were identified as highly significant (*p* < 0.01).

The results showed significant correlations between Zn and soil pH in the exchangeable and residual fractions (r_s_ = 0.78 and 0.79, respectively), indicating that Zn behavior is controlled by pH. The increased PMF values for Zn in samples SR3 and SR7 further support this claim. In addition, the significant correlation between Zn and Cd across all four extraction phases indicates their similar geochemical behavior. The Cr shows a strong correlation with Ni and Co in the residual fraction (r_s_ = 0.86 for both), indicating a similar lithogenic origin.

Although As and Pb have different distributions across the sequential extraction fractions, the high positive correlation between these two elements across all four fractions (r_s_ coefficients of 0.93, 0.93, 0.86, and 0.92 for the F1, F2, F3 and F4, respectively), suggests that, despite differences in their fractionation and dominant binding mechanisms, they share a similar spatial distribution (Figure 2), indicating a likely common source.

Negative correlations between Fe and As (r_s_ = −0.75) and Fe and Pb (r_s_ = −0.86) in the fourth phase further confirm that these elements are not part of the natural ferrous matrix. The predominance of As in the residual fraction may indicate that it formed insoluble crystals over time.

The strong positive correlations between Pb, As, and Cu in the first three phases of the extraction may indicate their common source. In contrast, the absence of correlations in the fourth phase suggests a different mode of soil stabilization.

Despite high concentrations of Pb in the reducible phase, the lack of significant correlations with Fe and Mn may indicate that Pb is mainly present in the form of secondary minerals, such as anglesite (PbSO_4_) or cerussite (PbCO_3_), suggesting an anthropogenic origin [60,62,69,73]. As previously discussed, the carbonate content in almost all samples is below the detection limit, indicating that Pb is present in the sulfate form. The high Pb concentrations and the presence of secondary minerals may provide a direct link to historical mining activities in this area.

### 3.5. Estimation of RAC and BF as Indicators of Environmental Risk

Environmental risk has previously been assessed using environmental and potential environmental risk indices; however, this approach has a significant limitation: both parameters rely on total element concentrations, which can be misleading and may overestimate the actual threat to the environment if the elements are bound in stable mineral phases. The Risk Assessment Code (RAC) and Bioaccessible Fraction (BF), by focusing on labile and bioavailable fractions, provide a more realistic assessment (Tables S8 and S9). These two factors are crucial for estimating the fraction of PTEs that can be easily released under changing soil conditions and pose a direct threat to the food chain. The Fe and Cr, with RAC values below 1%, are classified as posing no environmental risk. The Co, Ni, Zn, Mn, As, and Cu with RAC between 1% and 10% fall into the low-risk category. With the exception of sample SR7, Cd RAC values range from 11% to 25%, indicating a medium risk level. Sample SR7 shows a RAC of 32% for Cd, which is considered a high-risk category.

Because the largest portion of Pb is extracted in the second fraction, the RAC assessment classifies Pb as a low-environmental-risk element. Therefore, this low RAC value should be interpreted with appropriate caution. Its bioavailable fraction (BF) is significantly higher (ranging from 66% to 82%), indicating that Pb poses a potential ecological risk due to its sensitivity to environmental changes. Under conditions of decreasing soil pH and Eh, this “reserve” of pollutants could be rapidly remobilized, posing a direct threat to the environment [74,75]. Similarly, changes in soil conditions may cause Mn to contribute to secondary environmental pollution.

### 3.6. Health Risk Assessment

The PTE content pollution indices in the tested soil samples indicate a complex origin, involving both geogenic processes and anthropogenic inputs most likely related to historical mining activities in the study area. To assess potential impacts on human health, risk assessments were conducted for carcinogenic and non-carcinogenic effects from exposure to PTEs via three pathways: ingestion, dermal contact, and inhalation (Tables S26–S31) [57]. Hazard quotients (HQs) were estimated for each exposure pathway, and the results indicated that ingestion was the dominant route of exposure. Within the ingestion pathway, HQ values for individual PTE indicated that As contributed the most (54–78%) and Pb contributed the least (6–43%). HQ values for Pb in samples SR3 (2.04), SR6 (4.19), and SR7 (2.09) were above the acceptable threshold (HQ = 1), indicating an elevated non-carcinogenic health risk. Additionally, As HQs, except for SR4, exceeded the acceptable threshold, indicating a significant health risk. The total hazard index (HI_total_) was used to assess the overall non-carcinogenic risk at all sampling sites, including all investigated PTEs and all three exposure levels. The results showed that HI_total_ values exceeded 1 at most sampling sites, indicating an increased potential health risk. Observed results highlight the importance of assessing the cumulative risk to human health for all PTEs, without giving exclusive priority to a single exposure route.

The results show that the average cancer risk across all examined PTEs is below the acceptable limit for all exposure routes, except for As via ingestion. For the SR6 soil sample, the CRing for Pb is 1.25 × 10^−4^, which exceeds the permitted limit of 1 × 10^−4^. The obtained CRing values for As ranged from 1.03 × 10^−3^ (SR4) to 1.12 × 10^−2^ (SR3), exceeding the acceptable regulatory risk threshold of 1 × 10^−4^ across all analyzed samples. For comparison, using the As slope factor (SF = 1.5) from regulatory guidance prior to the 2025 EPA revision would yield substantially lower risk estimates, ranging from 5.25 × 10^−5^ (SR4) to 4.81 × 10^−4^ (RS3), emphasizing the risk assessment model’s high sensitivity to regulatory changes (Table S32). However, these results represent a worst-case scenario and may therefore be overestimated. Standard US EPA risk models assume 100% oral bioaccessibility of the element, whereas the BCR sequential extraction results indicate that up to 80% of As is bound to residual fractions. Although a mathematical bioaccessibility correction factor was not incorporated into the initial equations due to the lack of site-specific in vitro gastrointestinal extraction data, the fractionation data strongly suggest that the actual risk to human health associated with As exposure from the examined soil samples is significantly lower than the theoretical values indicate.

## 4. Conclusions

By combining the sequential extraction procedure with geochemical pollution indices, it was shown that elevated concentrations of potentially toxic elements in the studied soils are linked to both the natural lithogenic background of ore-rich parent material and the long-term anthropogenic impact of centuries of mining activities in the area. Although correlation analysis indicated that Pb, As, and Cd share a common origin in primary minerals, the high contents of Pb and Cd in most labile fractions, and of As in the residual fraction, suggest different stabilization in the soil. Ecological risk assessment indicates that Pb and, especially, Cd pose a potential ecological risk. These elements are sensitive to environmental changes and could be rapidly remobilized, becoming a direct threat to the environment. In contrast, the assessment of health risks for the local rural population, both carcinogenic and non-carcinogenic, showed that As made the largest contribution, primarily via ingestion. Therefore, assessing health risk at this site would require further studies to more precisely determine the proportion of this element that could be released under realistic gastrointestinal conditions and pose an actual health risk.

## Figures and Tables

**Table 1:** Descriptive statistics of analyzed soil parameters.

Parameters	Min	Mean	Median	Max	SD	CV (%)	UCC (mg/kg) *	CMAC ** (mg/kg)	CRC ** (mg/kg)
As	24.52	111.31	81.80	224.42	22.71	73.10	4.80	27.00	51.21
Cd	0.72	2.94	1.87	7.00	0.07	74.20	0.09	0.75	11.27
Co	17.22	21.94	21.49	25.64	2.33	13.80	17.00	7.85	209.39
Cr	48.32	78.70	69.53	141.11	30.86	46.40	92.00	91.80	348.84
Cu	40.09	51.45	51.03	71.46	8.92	21.80	28.00	33.00	174.17
Fe	41,319	49,945	48,378	55,643	4498	10.10	50,400	/	/
Mn	1103	1447	1339	2060	93.90	21.80	600.00	/	/
Ni	69.90	115.34	105.91	177.85	41.39	35.60	47.00	31.11	186.67
Pb	173.31	3187	1151	10,261	2810	119.60	17.00	80.00	498.82
Zn	141.77	409.15	314.52	724.82	37.91	52.30	67.00	140.00	720.00
pH (KCl) **	3.92	5.52	5.45	7.28	/		/	/	/
pH (H_2_O) **	5.20	6.29	6.38	8.00	/		/	/	/
CaCO_3_ (%) **	˂0.04	/	˂0.04	2.86	/	/	/	/	/
OM (%)	6.51	9.11	7.80	15.09	2.82	30.92	/	/	/

* The Upper Continental Crust (UCC) concentration, ** Corrected maximum allowable concentrations (CMAC) and corrected remediation concentrations (CMRC) in accordance with the Republic of Serbia Regulation standards (Table S2). ** SD and CV (%) were not calculated for pH values because of the logarithmic nature of the pH scale. For the carbonate fraction, Mean, SD, and CV (%) were not computed because the majority of the samples (5 out of 7) were below the limit of detection (<0.04%).

## Data Availability

The original contributions presented in this study are included in the article/Supplementary Material. Further inquiries can be directed to the corresponding author.

## Supplementary Materials

The following supporting information can be downloaded at: https://www.mdpi.com/article/10.3390/toxics14070608/s1, Table S1. Limits of detection (LOD) and limits of quantification (LOQ) for the investigated elements (mg/kg); Table S2. Validation ot the BCR sequential extraction procedure based on the recovery (%) of PTEs in soil samples (ΣF1-F4 vs. aqua regia concentrations); Table S3. Maximum available concentrations and remediation concentrations (mg/kg) and correction factors for CMAC and CRC calculation; Table S4. Ecological risk indices, the equations, and classification; Table S5. Ni, RAC, BF and BMF; Table S6. Ecological risk index (ERI); Table S7. Potential ecological risk index (PERI), Nemerov index (NI) and The Pollution Load Index (PLI); Table S8. Risk Assessment Code (RAC) values for PTE contamination in soil samples; Table S9. Potentially mobile fraction (PMF) of PTEs in soil samples (mg/kg); Table S10. Exposure parameters applied in the health risk assessment across various soil exposure pathways; Table S11. Reference doses (RfD) [mg/(kg*day)] and the cancer slope factors (CSF) [mg/(kg*day)] of PTEs for ingestion, dermal and inhalation pathways; Table S12. Equations for calculation of the health risk assessment model [75]; Table S13. Granulometric composition and soil textural class classification; Table S14. Concentration of PTEs (mg/kg) in soil samples determined after aqua regia digestion; Table S15. Enrichment Factor (EF); Table S16. Geoaccumulation Index (Igeo); Table S17. Concentration of PTEs (mg/kg) in the exchangeable BCR fraction (F1); Table S18. Concentration of PTEs (mg/kg) in the reducible BCR fraction (F2); Table S19. Concentration of PTEs (mg/kg) in the oxidizable BCR fraction (F3); Table S20. Concentration of PTEs (mg/kg) in the residual BCR fraction (F4); Table S21. Spearman’s rank correlation matrix for soil parameters in the studied area; Table S22. Spearman’s rank correlation matrix of soil parameters in the exchangeable phase of BCR sequential extraction; Table S23. Spearman’s rank correlation matrix of soil parameters in the reducible phase of BCR sequential extraction; Table S24. Spearman’s rank correlation matrix of soil parameters in the oxidizable phase of BCR sequential extraction; Table S25. Spearman’s rank correlation matrix of soil parameters in the residual phase of BCR sequential extraction; Table S26. Average daily dose (ADD) for oral exposure to PTEs; Table S27. Hazard Quotient (HQ) and Hazards Index for oral exposure to PTEs; Table S28. Average daily dose (ADD) for dermal exposure to PTEs; Table S29. Hazard Quotient (HQ) and Hazards Index for dermal exposure to PTEs; Table S30. Average daily dose (ADD) for inhalation exposure to PTEs; Table S31. Hazard Quotient (HQ) and Hazards Index for inhalation exposure to PTEs; Table S32. Estimated carcinogenic risk (CR) for As, Pb, Cd, and Ni via different exposure pathways. References [28,30,33,35,44,45,46,76,77,78,79,80,81,82,83,84,85,86,87,88,89,90,91,92,93] are cited in the Supplementary Materials.
