# Supplementary material for: Potentially Toxic Elements in Soil of a Historical Mining Region in Serbia: Geochemical Fractionation, Ecological Impact, and Health Risk Assessment
Source: Toxics. 2026 Jul 12;14(7):608. doi: 10.3390/toxics14070608 (PMC13417382; doi:10.3390/toxics14070608)
Supplement: Supplementary file 1 [file toxics-14-00608-s001.zip › toxics-4393811-supplementary.pdf]

Supplementary material

Potentially Toxic Elements in Soil of a Historical Mining Region in Serbia: Geochemical Fractionation, Ecological Impact, and Health Risk Assessment

Almasa Lekpek[1], Svetlana Đogo Mračević[2,*], Zoran Dinić[3], Aleksandra Šajnović[4], Slavica Ražić[2], Milan Stanković[2], Branimir Jovančićević[1]

[1] Faculty of Chemistry, University of Belgrade, Studentski trg 12-16, 11000 Belgrade, Serbia;
[2] Faculty of Pharmacy, University of Belgrade, Vojvode Stepe 450, 11221 Belgrade, Serbia;
[3] Institute of Soil Science, Teodora Drajzera 7, 11000 Belgrade, Serbia;
[4] Institute of Chemistry, Technology and Metallurgy, University of Belgrade, Njegoševa 12, 11000 Belgrade, Serbia
* Corresponding author: svetlana.djogo@pharmacy.bg.ac.rs

Table S1. Limits of detection (LOD) and limits of quantification (LOQ) for the investigated elements (mg/kg)

Elements	LOD	LOQ
As	0.01	0.03
Cd	0.01	0.03
Co	0.01	0.03
Cr	0.03	0.09
Cu	0.03	0.09
Fe	0.05	0.15
Mn	0.05	0.15
Ni	0.04	0.12
Pb	0.06	0.18
Zn	0.05	0.15

Table S2. Validation ot the BCR sequential extraction procedure based on the recovery (%) of PTEs in soil samples (ΣF1-F4 vs. aqua regia concentrations)

Sampling site	As	Cd	Co	Cr	Cu	Fe	Mn	Ni	Pb	Zn
SR1	99.13	110.56	114.19	106.63	101.73	87.68	114.08	110.11	101.01	105.63
SR2	104.61	115.46	119.39	112.64	119.66	95.07	118.23	114.78	109.28	110.15
SR3	93.25	100	118.11	111.56	94.8	95.44	117.1	119.48	84.22	116.96
SR4	117.13	109.15	119.28	118.18	117.46	103.12	117.67	119.45	106.32	119.66
SR5	113.62	111.7	118.42	117.31	112.86	99.01	111.11	117.6	115.4	119.76
SR6	112.9	101.7	115.48	118.82	117.36	103.37	117.94	119.37	82.97	106.28
SR7	93.67	119.92	117.02	99.41	97.77	81.08	117.62	103.66	110.01	105.15
Max	117.13	119.92	119.39	118.82	119.66	103.37	118.23	119.48	115.4	119.76
Min	93.25	100	114.19	99.41	94.8	81.08	111.11	103.66	82.97	105.15
Mean	104.9	109.78	117.41	112.08	108.81	94.97	116.25	114.92	101.31	111.94

Table S3. Maximum available concentrations and remediation concentrations (mg/kg) and correction factors for CMAC and CRC calculation

Elements	MAC	RC	A	B	C
As	29.00	55.00	15.00	0.40	0.40
Cd	0.80	12.00	0.40	0.01	0.02
Co	9.00	240.00	2.00	0.28	0.00
Cr	100.00	380.00	50.00	2.00	0.00
Cu	36.00	190.00	15.00	0.60	0.60
Ni	35.00	210.00	10.00	1.00	1.00
Pb	85.00	530.00	50.00	1.00	1.00

MAC – maximum available concentration; RC – remediation concentration; CMAC – corrected maximum available concentration, CRC – corected remediation concentration,

The equation for calculating CMAC and CRC values [28]:

$$(CMAC, CRC) = (MAC, RC) \times (A + B \times \%\,Clay + (C \times \%\,OM)/(A + B \times 25 + C \times 10)$$

The percentage of clay and organic matter (OM) represents mean values of the measured results (Tables S14 and S17).

Table S4. Ecological risk indices, the equations, and classification.

Index; Range of values	Equation; Classifications description
Enrichment factor	$EF = [(C_x/R)_{sample}]/[(C_x/R)_{reference}]$ [76]
EF < 2	- deficiency to minimal enrichment
2 ≤ EF < 5	- moderate enrichment
5 ≤ EF < 20	- significant enrichment
20 ≤ EF < 40	- very high enrichment
EF ≥ 40	- extremely high enrichment
Geoaccumulation index	$I_{geo} = \log_2[C_x/(1.5 \times B_n)]$ [76]
$I_{geo} < 0$	- uncontaminated (Class 0)
$0 < I_{geo} < 1$	- uncontaminated to moderately contaminated (Class 1)
$1 \leq I_{geo} < 2$	- moderately contaminated (Class 2)
$2 \leq I_{geo} < 3$	- moderately to strongly contaminated (Class 3)
$3 \leq I_{geo} < 4$	- strongly contaminated (Class 4)
$4 \leq I_{geo} < 5$	- strongly to extremely contaminated (Class 5)
$I_{geo} \geq 5$	- extremely contaminated (Class 6)
Nemerov Index	$NI = \sqrt{I_{geo}average^2 + I_{geo}max^2}$ [30]
NI ≤ 0	- uncontaminated soils
0 < NI ≤ 1	- slightly contaminated soils
1 < NI ≤ 2	- moderately contaminated soils
2 < NI ≤ 3	- highly contaminated soils
NI > 3	- very highly contaminated soils
The Pollution Load Index	$PLI = \sqrt[n]{(CF1 * CF2 * CF3 * \ldots * CFn)}$ [77]
PLI < 1	- uncontaminated
1 ≤ PLI < 2	-uncontaminated to moderately contaminated
2 ≤ PLI < 3	- moderately to strongly contaminated
PLI ≥ 3	- strongly contaminated

Ecological Risk Index	$ERI = Ti \times Cf$ [30]
ERI < 40	- no ecological risk
40 < ERI ≤ 80	- general ecological risk
80 < ERI ≤ 160	- moderate ecological risk
160 < ERI ≤ 320	- high ecological risk
ERI > 320	- severe ecological risk
Potential Risk Index	PERI = Σ ($T_i \times C_x / B_n$) [76]
PERI < 150	- low ecological risk
150 ≤ PERI < 300	- moderate ecological risk
300 ≤ PERI < 600	- significant ecological risk
PERI ≥ 600	- very high ecological risk.

Cx - represents the concentration of individual element; R – represents the concentration of referent element Fe; B_n - is the element concentration in the reference (UCC) sample; I_{geo} – geo-accumulation index; n - the total number of heavy metals or pollutants being studied; CF - the contamination factor for each individual pollutant; T*i* – a coefficient designed to evaluate the relative toxicity and environmental sensitivity of a specific element; Cf - the ratio of the measured concentration of a specific contaminant to its regional or natural background baseline concentration

Table S5. N_i, RAC, BF and BMF

Index	Equation
*N_i	$Ni = C_x \, / \, C_x \, UCC$ [78]
**RAC	$RAC = F1 \, / \, (F1 + F2 + F3 + F4)$ [33,35]
***BF	$BF = (F1 + F2) \, / \, (F1 + F2 + F3 + F4)$ [35]
****PMF	$PMF = (F1 + F2 + F3) \, / \, (F1 + F2 + F3 + F4)$ [33,35]

* N_i - normalization factor; ** RAC - risk assessment code; *** BF - bioavailability factor;**** PMF – potentially mobile fraction; F1 – exchangeable fraction; F2 – reducible fraction; F3 – oxidizable fraction and F4 – residual fraction

Table S6. Ecological risk index (ERI)

Sampling site	As	Cd	Co	Cr	Cu	Fe	Mn	Ni	Pb	Zn
SR1	170.41	589.71	6.89	2.55	7.81	1.09	2.01	17.54	338.65	3.89
SR2	106.14	240.40	7.41	3.07	7.16	1.06	1.84	18.92	68.06	2.12
SR3	467.55	1571.81	5.73	1.51	12.76	0.95	2.57	11.13	1473.51	8.31
SR4	51.09	582.04	6.21	1.08	7.28	0.95	2.23	8.29	50.97	4.09
SR5	127.18	2332.03	7.22	1.05	9.11	1.10	3.43	11.27	104.54	10.82
SR6	463.50	919.68	4.98	1.12	10.12	0.82	2.58	7.44	3017.83	8.82
SR7	237.35	622.19	5.94	1.60	10.07	0.96	2.23	11.31	1507.58	4.69
Max	467.55	2332.03	7.41	3.07	12.76	1.10	3.43	18.92	3017.83	10.82
Min	51.09	240.40	4.98	1.05	7.16	0.82	1.84	7.44	50.97	2.12
Mean	231.89	979.69	6.34	1.71	9.19	0.99	2.41	12.27	937.31	6.11
SD	156.96	672.69	0.81	0.73	1.85	0.09	0.49	4.04	1037.47	2.96

Table S7. Potential ecological risk index (PERI), Nemerov index (NI) and The Pollution Load Index (PLI)

Sampling site	NI	PLI	PERI
SR1	5.70	4.23	1140.55
SR2	3.34	3.00	456.17
SR3	7.88	6.11	3555.83
SR4	3.78	2.60	714.25
SR5	5.90	4.39	2607.75
SR6	8.85	5.53	4436.87
SR7	7.83	4.80	2403.92
Max	8.85	6.11	4436.87
Min	3.34	2.60	456.17
Mean	6.18	4.38	2187.91

Table S8. Risk Assessment Code (RAC) values for PTEs contamination in soil samples

Sampling site	As	Cd	Co	Cr	Cu	Fe	Mn	Ni	Pb	Zn
SR1	1.32	23.82	3.47	0.03	2.33	0.05	9.47	1.49	5.29	5.36
SR2	0.74	20.48	4.25	0.05	0.74	0.05	10.89	1.09	2.36	2.35
SR3	0.36	22.68	1.89	0.00	1.11	0.03	4.29	1.04	6.24	5.47
SR4	0.26	10.96	1.95	0.02	0.63	0.10	4.48	2.04	2.07	1.46
SR5	0.28	23.49	1.99	0.00	1.11	0.03	7.18	4.21	0.59	3.00
SR6	0.45	25.17	2.34	0.00	2.99	0.09	3.94	2.85	5.46	4.44
SR7	1.21	32.32	2.11	0.01	4.01	0.07	5.14	2.20	10.04	8.76
Max	1.32	32.32	4.25	0.05	4.01	0.10	10.89	4.21	10.04	8.76
Min	0.26	10.96	1.89	0.00	0.63	0.03	3.94	1.04	0.59	1.46
Mean	0.66	22.70	2.57	0.02	1.85	0.06	6.49	2.13	4.58	4.41

Table S9. Potentially mobile fraction (PMF) of PTEs in soil samples (mg/kg)

Sampling site	As	Cd	Co	Cr	Cu	Fe	Mn	Ni	Pb	Zn
SR1	11.34	1.41	12.83	6.08	4.21	2003.36	1164.79	13.54	1039.60	52.45
SR2	7.24	0.49	14.96	7.49	1.72	2100.43	1072.72	10.44	215.30	13.42
SR3	22.44	3.55	10.34	3.52	9.40	1858.32	1386.71	10.22	3724.53	165.56
SR4	1.00	1.14	14.29	4.53	1.50	1882.78	1313.07	12.41	131.26	24.47
SR5	2.22	6.59	18.27	4.64	3.15	1820.83	2031.58	34.60	307.08	123.01
SR6	23.16	1.78	11.13	4.58	7.87	2256.56	1383.06	8.56	7255.06	87.56
SR7	21.97	1.79	10.50	6.53	10.71	2885.98	1304.44	10.85	5052.14	92.89
Max	23.16	6.59	18.27	7.49	10.71	2885.98	2031.58	34.60	7255.06	165.56
Min	1.00	0.49	10.34	3.52	1.50	1820.83	1072.72	8.56	131.26	13.42
Mean	12.77	2.39	13.19	5.34	5.51	2115.47	1379.48	14.37	2532.14	79.91

Table S10. Exposure parameters applied in the health risk assessment across various soil exposure pathways

Exposure parameter	Abbrev.	Units	Values	Ref.
Element concentration	C	mg*kg^{-1}	/	/
Ingestion rate	IngR	mg*day^{-1}	100	[79]
Inhalation rate	InhR	m3*day^{-1}	20	[80]
Conversion factor	CF	kg*mg^{-1}	1.00E-06	[30]
Body weight	BW	kg	70	[79]
Exposure frequency	EF	day(s)*year^{-1}	180	This study
Exposure duration	ED	year(s)	30	[79]
Skin surface area	SA	cm^2	5700	[79]
Adherence factor	AF	mg*cm^{-2}*day^{-1}	0.07	[81]
Dermal absorption factor	ABS_{derm}	/	0.01	[81]
Particle emission factor	PEF	m^3*kg^{-1}	1.36E+09	[81]
Average time	AT	days	365*ED	This study

Table S11. Reference doses (RfD) [mg/(kg*day)] and the cancer slope factors (CSF) [mg/(kg*day)] of PTEs for ingestion, dermal and inhalation pathways.

PTEs	RfD_{oral} [mg/(kg*day)]	Ref.	CSF_{oral} [mg/(kg*day)]	Ref.	ABS_{GI}	Ref.	RfD_{dermal} [mg/(kg*day)]	CSF_{dermal} [mg/(kg*day)]	Ref.	RfD_{inhal} [$mg \cdot m^{-3}$]	Ref.	CSF_{inhal} [mg/(kg*day)]	Ref.
As	3.00E-04	[82]	1.5 and 32	[44,90]	0.95	[92]	2.85E-04	1.50	[91]	1.00E-03	[78]	12.00	[76]
Cd	1.00E-03	[46]	0.38	[91]	0.08	[92]	8.00E-05	6.30	[91]	1.00E-03	[81]	6.30	[76]
Co	1.40E-01	[83]	N/A	N/A	0.25	[93]	3.50E-02	N/A	N/A	5.71E-06	[81]	N/A	N/A
Cr	3.00E-03	[84]	0.50	[84]	0.013	[93]	3.90E-05	N/A	N/A	2.86E-05	[81]	N/A	N/A
Cu	4.00E-02	[84]	N/A	N/A	0.30	[92]	1.20E-02	N/A	N/A	4.02E-02	[81]	N/A	N/A
Fe	7.00E-01	[85]	N/A	N/A	0.014	[90]	9.80E-03	N/A	N/A	N/A	[81]	N/A	N/A
Mn	3.00E-02	[86]	N/A	N/A	0.04	[93]	1.20E-03	N/A	N/A	1.43E-05	[78]	N/A	N/A
Ni	2.00E-02	[87]	0.84	[91]	0.04	[93]	8.00E-04	0.84	[91]	2.06E-02	[81]	0.91	[91]
Pb	3.50E-03	[88]	0.0085	[90]	1.00	[93]	3.50E-03	0.042	[91]	3.52E-03	[81]	0.042	[76]
Zn	3.00E-01	[89]	N/A	N/A	0.61	[92]	1.83E-01	N/A	N/A	3.00E-01	[81]	N/A	N/A

* N/A – not available

Table S12. Equations for calculation of the health risk assessment model [93].

Average Daily Dose

$$ADD_{i,ing} = \frac{C \times IngR \times EF \times ED}{BW \times AT} \times CF$$

$$ADD_{i,inh} = \frac{C \times InhR \times EF \times ED}{PEF \times BW \times AT}$$

$$ADD_{i,derm} = \frac{C \times SA \times AF \times ABSderm \times EF \times ED}{BW \times AT} \times CF$$

Non-Carcinogenic Risk

$$HQ_{i,inh} = \frac{ADD_{i,inh}}{RfD_{i,inh}}$$

$$HQ_{i,derm} = \frac{ADD_{i,derm}}{RfD_{i,derm}}$$

$$HQ_i = HQ_{i,ing} + HQ_{i,inh} + HQ_{i,derm}$$

$$HI = \sum_{i=1}^{n} HQ_i$$

Carcinogenic Risk

$$CSF_{i,derm} = \frac{CSF_{i,ing}}{ABS_{GI}}$$

$$CR_{i,ing} = ADD_{i,ing} \times CSF_{i,ing}$$

$$CR_{i,inh} = ADD_{i,inh} \times CSF_{i,inh}$$

$$CR_{i,derm} = ADD_{i,derm} \times CSF_{i,derm}$$

$$CR_i = CR_{i,ing} + CR_{i,inh} + CR_{i,derm}$$

$$TCR = \sum_{i=1}^{n} CR_i$$

*RfD – reference dose; TCR – total carcinogenic risk

Table S13. Granulometric composition and soil textural class classification

Sampling site	Depth (cm)	Coarse sand > 0.2 mm (%)	Fine sand 0.2 - 0.02 mm (%)	Silt 0.02-0.002 mm (%)	Clay < 0.002 mm (%)	Total sand > 0.02 mm (%)	Fraction under 2 mm (%)	Fraction over 2 mm (%)	Texture Class	Soil type
SR1	0-30	26.8	26.2	29.1	17.9	53.0	66.84	33.16	Clay loam	Leptosols
SR2	0-30	23.6	23.3	28.1	25.0	46.9	57.58	42.42	Clay loam	Leptosols
SR3	0-30	34.2	24.3	24.0	17.5	58.5	63.34	36.66	Clay loam	Fluvisols
SR4	0-30	19.7	23.3	33.7	23.3	43.0	47.58	52.42	Clay loam	Leptosols
SR5	0-30	28.5	21.6	25.9	24.0	50.1	48.98	51.02	Clay loam	Leptosols
SR6	0-30	28.7	25.3	27.6	18.4	54.0	58.74	41.26	Clay loam	Leptosols
SR7	0-30	31.4	23.5	25.1	20.0	54.9	58.10	41.90	Clay loam	Fluvisols

Table S14. Concentration of PTEs (mg/kg) in soil samples determined after aqua regia digestion

Sampling site	As	Cd	Co	Cr	Cu	Fe	Mn	Ni	Pb	Zn	pH (KCl)	pH (H_2O)	Carbonates(%)	OM (%)
SR1	81.80	1.77	23.84	117.18	43.76	54739.30	1204.36	164.87	1151.42	260.90	5.45	6.38	< 0.04	7.57
SR2	50.95	0.72	25.64	141.11	40.09	53513.80	1102.79	177.85	231.40	141.77	4.60	5.65	< 0.04	10.10
SR3	224.42	4.72	19.84	69.53	71.46	47948.38	1540.94	104.59	5009.93	557.08	7.28	8.00	2.86	6.51
SR4	24.52	1.75	21.49	49.64	40.78	48076.60	1338.50	77.96	173.31	274.19	3.92	5.20	< 0.04	7.80
SR5	61.04	7.00	24.98	48.32	51.03	55642.50	2060.45	105.91	355.42	724.82	5.53	6.52	< 0.04	7.77
SR6	222.48	2.76	17.22	51.59	56.65	41319.31	1547.81	69.90	10260.62	590.77	4.52	5.41	< 0.04	8.91
SR7	113.93	1.87	20.54	73.53	56.38	48377.97	1337.16	106.33	5125.77	314.52	6.36	6.90	0.09	15.09

Table S15. Enrichment Factor (EF)

Sampling site	As	Cd	Co	Cr	Cu	Mn	Ni	Pb	Zn
SR1	15.69	18.10	1.27	55.75	1.44	1.85	3.23	62.36	3.59
SR2	10.00	7.55	1.40	1.44	1.35	1.73	3.56	12.82	1.99
SR3	49.15	55.07	1.21	0.79	2.68	2.70	2.34	309.77	8.74
SR4	5.36	20.34	1.30	0.57	1.53	2.34	1.74	10.69	4.29
SR5	11.52	70.41	1.31	0.48	1.65	3.11	2.04	18.94	9.8
SR6	56.54	37.39	1.21	0.68	2.47	3.15	1.81	736.21	10.76
SR7	24.73	21.61	1.24	0.83	2.10	2.32	2.36	314.12	4.89
Max	56.54	70.41	1.40	55.75	2.68	3.15	3.56	736.21	10.76
Min	5.36	7.55	1.21	0.48	1.35	1.73	1.74	10.69	1.99
Mean	24.71	32.92	1.28	8.65	1.89	2.46	2.44	209.27	6.29
SD	18.73	20.93	0.06	19.23	0.49	0.52	0.65	249.17	3.16

Table S16. Geoaccumulation Index (I_{geo})

Sampling site	As	Cd	Co	Cr	Cu	Fe	Mn	Ni	Pb	Zn
SR1	3.51	3.71	-0.12	-0.24	0.06	-0.47	0.42	1.23	5.50	1.38
SR2	2.82	2.42	-0.02	0.03	-0.07	-0.5	0.29	1.33	3.18	0.5
SR3	4.96	5.13	-0.39	-0.99	0.77	-0.66	0.78	0.57	7.62	2.47
SR4	1.77	3.69	-0.27	-1.48	-0.04	-0.65	0.57	0.15	2.76	1.45
SR5	3.08	5.70	-0.05	-1.51	0.28	-0.44	1.19	0.59	3.80	2.85
SR6	4.95	4.35	-0.59	-1.42	0.43	-0.87	0.78	-0.01	8.65	2.56
SR7	3.98	3.79	-0.34	-0.91	0.42	-0.64	0.57	0.59	7.65	1.65
Max	0.29	0.78	0.57	1.19	0.78	0.57	1.19	1.33	8.65	2.85
Min	1.33	0.57	0.15	0.59	-0.01	0.59	0.29	-0.01	2.76	0.50
Mean	3.18	7.62	2.76	3.8	8.65	7.65	0.66	0.63	5.60	1.83
SD	0.50	2.47	1.45	2.85	2.56	1.65	0.27	0.46	2.23	0.77

Table S17. Concentration of PTEs (mg/kg) in the exchangeable BCR fraction (F1)

Sampling site	As	Cd	Co	Cr	Cu	Fe	Mn	Ni	Pb	Zn
SR1	1.07	0.47	0.94	0.03	1.04	24.10	130.11	2.70	61.54	14.77
SR2	0.39	0.17	1.30	0.08	0.36	23.43	142.00	2.23	5.96	3.67
SR3	0.75	1.07	0.44	< LOD	0.75	11.95	77.43	1.30	263.43	35.65
SR4	0.07	0.21	0.50	0.01	0.30	49.32	70.60	1.90	3.82	4.80
SR5	0.19	1.84	0.59	< LOD	0.64	15.15	164.42	5.24	2.42	26.08
SR6	1.12	0.71	0.47	< LOD	1.99	37.92	71.95	2.38	464.82	27.90
SR7	1.29	0.72	0.51	< LOD	2.21	28.50	80.80	2.43	565.99	28.96
Max	1.29	1.84	1.30	0.08	2.21	49.32	164.42	5.24	565.99	35.65
Min	0.07	0.17	0.44	< LOD	0.30	11.95	70.60	1.30	2.42	3.67
Mean	0.70	0.74	0.68	0.02	1.04	27.20	105.33	2.60	195.43	20.26
SD	0.16	0.18	0.30	0.02	0.83	3.11	34.87	0.19	356.70	10.03

Table S18. Concentration of PTEs (mg/kg) in the reducible BCR fraction (F2)

Sampling site	As	Cd	Co	Cr	Cu	Fe	Mn	Ni	Pb	Zn
SR1	5.08	0.86	11.31	1.06	1.11	1332.00	1008.70	6.23	895.12	27.00
SR2	2.50	0.28	12.87	1.12	0.34	1308.87	893.50	3.53	184.26	6.14
SR3	9.11	2.18	9.17	0.82	1.86	1334.63	1263.99	6.31	3022.98	85.51
SR4	0.19	0.79	13.10	0.75	0.54	1444.58	1195.14	6.67	117.63	12.04
SR5	0.51	4.51	16.91	1.04	0.78	1305.79	1822.01	23.39	273.61	60.54
SR6	9.88	0.90	10.10	0.92	1.93	1582.49	1270.05	3.68	5906.74	36.50
SR7	10.87	0.92	9.27	1.06	1.54	1671.74	1195.71	3.53	3981.64	40.49
Max	10.87	4.51	16.91	1.12	1.93	1671.74	1822.01	23.39	5906.74	85.51
Min	0.19	0.28	9.17	0.75	0.34	1305.79	893.50	3.53	117.63	6.14
Mean	5.45	1.49	11.82	0.97	1.16	1425.73	1235.59	7.62	2054.57	38.32
SD	4.09	0.04	1.44	0.00	0.30	240.23	132.24	1.91	2182.50	9.54

Table S19. Concentration of PTEs (mg/kg) in the oxidizable BCR fraction (F3)

Sampling site	As	Cd	Co	Cr	Cu	Fe	Mn	Ni	Pb	Zn
SR1	5.19	0.08	0.57	4.99	2.07	647.26	25.98	4.61	82.95	10.68
SR2	4.35	0.04	0.79	6.29	1.03	768.13	37.22	4.68	25.07	3.61
SR3	12.58	0.30	0.73	2.69	6.79	511.74	45.28	2.60	438.12	44.39
SR4	0.74	0.14	0.70	3.77	0.66	388.88	47.33	3.84	9.80	7.63
SR5	1.52	0.25	0.78	3.60	1.73	499.89	45.16	5.97	31.05	36.39
SR6	12.16	0.17	0.57	3.66	3.95	636.15	41.06	2.50	883.50	23.16
SR7	9.81	0.14	0.72	5.46	6.95	1185.74	27.92	4.89	504.52	23.44
Max	12.58	0.30	0.79	6.29	6.95	1185.74	47.33	5.97	883.50	44.39
Min	0.74	0.04	0.57	2.69	0.66	388.88	25.98	2.50	9.80	3.61
Mean	6.62	0.16	0.69	4.35	3.31	662.54	38.56	4.16	282.14	21.33
SD	3.27	0.04	0.11	0.33	3.45	380.76	1.37	0.20	298.10	9.02

Table S20. Concentration of PTEs (mg/kg) in the residual BCR fraction (F4)

Sampling site	As	Cd	Co	Cr	Cu	Fe	Mn	Ni	Pb	Zn
SR1	69.75	0.55	14.40	118.87	40.31	45990.01	209.20	167.99	123.47	223.14
SR2	46.06	0.34	15.65	151.47	46.26	48774.61	231.10	193.69	37.57	142.74
SR3	186.83	1.17	13.10	74.05	58.34	43901.56	417.75	114.74	494.77	485.99
SR4	27.73	0.77	11.34	54.14	46.41	47691.71	261.90	80.71	53.01	303.64
SR5	67.14	1.22	11.31	52.05	54.45	53272.81	257.80	89.95	103.07	745.04
SR6	228.03	1.03	8.76	56.72	58.62	40456.51	442.45	74.88	1258.37	540.34
SR7	84.75	0.45	13.54	66.58	44.42	36339.11	268.30	99.37	586.47	237.84
Max	228.03	1.22	15.65	151.47	58.62	53272.81	442.45	193.69	1258.37	745.04
Min	27.73	0.34	8.76	52.05	40.31	36339.11	209.20	74.88	37.57	142.74
Mean	101.47	0.79	12.58	81.98	49.83	45203.76	298.36	117.33	379.53	382.67
SD	10.61	0.07	0.61	36.97	2.91	6824.22	41.79	48.52	327.39	10.39

Table S21. Spearman's rank correlation matrix for soil parameters in the studied area

	As	Cd	Co	Cr	Cu	Fe	Mn	Ni	Pb	Zn	Clay	OM	pH
**As**													
**Cd**	0.61												
**Co**	-0.75*	-0.39											
**Cr**	0.07	-0.61	0.25										
**Cu**	0.93**	0.79*	-0.82*	-0.29									
**Fe**	-0.50	-0.04	0.86**	0.11	-0.54								
**Mn**	0.32	0.86**	-0.43	-0.89**	0.61	-0.21							
**Ni**	-0.29	-0.43	0.75*	0.75*	-0.54	0.71	-0.75*						
**Pb**	0.89**	0.50	-0.75*	0.07	0.82*	-0.46	0.29	-0.29					
**Zn**	0.46	0.93**	-0.46	-0.79*	0.71	-0.18	0.96**	-0.64	0.46				
**Clay**	-0.79*	-0.36	0.71	-0.07	-0.71	0.46	-0.14	0.32	-0.61	-0.18			
**OM**	-0.25	-0.43	0.07	0.25	-0.29	-0.07	-0.32	0.18	0.11	-0.21	0.57		
**pH**	0.61	0.57	-0.11	0.14	0.57	0.18	0.11	0.29	0.39	0.29	-0.39	-0.29	

** $p < 0.01$; * $p < 0.05$. Spearman's correlation coefficients are shown. Diagonal elements (1.00) are omitted for clarity.

Table S22. Spearman's rank correlation matrix of soil parameters in the exchangeable phase of BCR sequential extraction

	As	Cd	Co	Cr	Cu	Fe	Mn	Ni	Pb	Zn	Clay	OM	pH
**As**													
**Cd**	0.18												
**Co**	-0.14	-0.43											
**Cr**	-0.15	-0.85*	0.74										
**Cu**	0.96**	0.39	-0.21	-0.33									
**Fe**	0.11	-0.50	-0.11	0.22	0.07								
**Mn**	-0.07	0.21	0.75*	0.19	-0.04	-0.64							
**Ni**	0.21	0.32	0.54	-0.04	0.36	-0.04	0.64						
**Pb**	0.93**	0.11	-0.39	-0.19	0.86*	0.18	-0.36	-0.14					
**Zn**	0.57	0.79*	-0.71	-0.78*	0.68	-0.29	-0.21	-0.07	0.64				
**Clay**	-0.50	-0.32	0.61	0.37	-0.54	0.11	0.43	0.21	-0.57	-0.68			
**OM**	0.32	-0.36	0.29	0.30	0.21	0.50	0.00	0.11	0.32	-0.21	0.57		
**pH**	0.36	0.71	-0.14	-0.37	0.43	-0.75*	0.39	0.11	0.32	0.71	-0.39	-0.29	

** $p < 0.01$; * $p < 0.05$. Spearman's correlation coefficients are shown. Diagonal elements (1.00) are omitted for clarity.

Table S23. Spearman's rank correlation matrix of soil parameters in the reducible phase of BCR sequential extraction

	As	Cd	Co	Cr	Cu	Fe	Mn	Ni	Pb	Zn	Clay	OM	pH
**As**													
**Cd**	0.29												
**Co**	-0.86*	-0.21											
**Cr**	0.29	-0.21	0.00										
**Cu**	0.79*	0.54	-0.75*	-0.29									
**Fe**	0.64	-0.04	-0.61	-0.21	0.57								
**Mn**	0.18	0.86*	-0.04	-0.43	0.57	0.07							
**Ni**	-0.71	0.36	0.54	-0.68	-0.18	-0.50	0.43						
**Pb**	0.93**	0.46	-0.75*	0.07	0.93**	0.57	0.46	-0.46					
**Zn**	0.39	0.96**	-0.43	-0.29	0.64	0.07	0.79*	0.29	0.54				
**Clay**	-0.54	-0.32	0.75*	0.39	-0.79*	-0.39	-0.18	0.00	-0.61	-0.50			
**OM**	0.29	-0.36	0.04	0.54	-0.18	0.43	-0.21	-0.71	0.11	-0.43	0.57		
**pH**	0.46	0.71	-0.54	0.21	0.36	-0.07	0.29	-0.07	0.39	0.79*	-0.39	-0.29	

** $p < 0.01$; * $p < 0.05$. Spearman's correlation coefficients are shown. Diagonal elements (1.00) are omitted for clarity.

Table S24. Spearman's rank correlation matrix of soil parameters in the oxidizable phase of BCR sequential extraction

	As	Cd	Co	Cr	Cu	Fe	Mn	Ni	Pb	Zn	Clay	OM	pH
**As**													
**Cd**	0.46												
**Co**	-0.25	0.00											
**Cr**	-0.29	-0.89**	0.11										
**Cu**	0.86*	0.50	-0.21	-0.18									
**Fe**	0.39	-0.43	0.07	0.71	0.50								
**Mn**	-0.21	0.50	0.14	-0.64	-0.36	-0.86*							
**Ni**	-0.50	-0.18	0.61	0.36	-0.11	0.25	-0.32						
**Pb**	0.86*	0.46	-0.46	-0.21	0.89**	0.43	-0.36	-0.32					
**Zn**	0.54	0.93**	0.07	-0.75*	0.68	-0.18	0.21	0.04	0.54				
**Clay**	-0.75*	-0.43	0.57	0.46	-0.64	0.00	0.07	0.61	-0.61	-0.50			
**OM**	-0.14	-0.43	0.07	0.71	0.04	0.57	-0.32	0.29	0.11	-0.43	0.57		
**pH**	0.54	0.54	0.39	-0.29	0.71	0.29	-0.18	0.32	0.39	0.79*	-0.39	-0.29	

** $p < 0.01$; * $p < 0.05$. Spearman's correlation coefficients are shown. Diagonal elements (1.00) are omitted for clarity

Table S25. Spearman's rank correlation matrix of soil parameters in the residual phase of BCR sequential extraction

	As	Cd	Co	Cr	Cu	Fe	Mn	Ni	Pb	Zn	Clay	OM	pH
**As**													
**Cd**	0.29												
**Co**	-0.32	-0.79*											
**Cr**	0.07	-0.68	0.86*										
**Cu**	0.43	0.71	-0.79*	-0.46									
**Fe**	-0.75*	0.18	0.07	-0.14	-0.04								
**Mn**	0.68	0.39	-0.64	-0.36	0.75*	-0.64							
**Ni**	-0.21	-0.54	0.93**	0.86*	-0.61	0.21	-0.64						
**Pb**	0.93**	0.25	-0.43	-0.14	0.32	-0.86*	0.71	-0.43					
**Zn**	0.36	0.93**	-0.93**	-0.82*	0.79*	0.04	0.57	-0.75*	0.39				
**Clay**	-0.71	-0.29	0.11	-0.18	-0.14	0.64	-0.39	0.04	-0.68	-0.14			
**OM**	-0.11	-0.64	0.14	0.04	-0.21	-0.25	0.11	-0.11	0.04	-0.32	0.57		
**pH**	0.43	0.21	0.21	0.18	-0.07	-0.25	0.11	0.39	0.32	0.11	-0.39	-0.29	

** $p < 0.01$; * $p < 0.05$. Spearman's correlation coefficients are shown. Diagonal elements (1.00) are omitted for clarity.

Table S26. Average daily dose (ADD) for oral exposure to PTEs

Sampling site	Elements									
	As	Cd	Co	Cr	Cu	Fe	Mn	Ni	Pb	Zn
SR1	1.17E-04	2.53E-06	3.41E-05	1.67E-04	6.25E-05	7.82E-02	1.72E-03	2.36E-04	1.64E-03	3.73E-04
SR2	7.28E-05	1.03E-06	3.66E-05	2.02E-04	5.73E-05	7.64E-02	1.58E-03	2.54E-04	3.31E-04	2.03E-04
SR3	3.21E-04	6.74E-06	2.83E-05	9.93E-05	1.02E-04	6.85E-02	2.20E-03	1.49E-04	7.16E-03	7.96E-04
SR4	3.50E-05	2.49E-06	3.07E-05	7.09E-05	5.83E-05	6.87E-02	1.91E-03	1.11E-04	2.48E-04	3.92E-04
SR5	8.72E-05	9.99E-06	3.57E-05	6.90E-05	7.29E-05	7.95E-02	2.94E-03	1.51E-04	5.08E-04	1.04E-03
SR6	3.18E-04	3.94E-06	2.46E-05	7.37E-05	8.09E-05	5.90E-02	2.21E-03	9.99E-05	1.47E-02	8.44E-04
SR7	1.63E-04	2.67E-06	2.93E-05	1.05E-04	8.05E-05	6.91E-02	1.91E-03	1.52E-04	7.32E-03	4.49E-04
Max	3.21E-04	9.99E-06	3.66E-05	2.02E-04	1.02E-04	7.95E-02	2.94E-03	2.54E-04	1.47E-02	1.04E-03
Min	3.50E-05	1.03E-06	2.46E-05	6.90E-05	5.73E-05	5.90E-02	1.58E-03	9.99E-05	2.48E-04	2.03E-04
Mean	1.59E-04	4.20E-06	3.13E-05	1.12E-04	7.35E-05	7.14E-02	2.07E-03	1.65E-04	4.55E-03	5.85E-04

Table S27. Hazard Quotient (HQ) and Hazards Index for oral exposure to PTEs

Sampling site	HQ										HI
	As	Cd	Co	Cr	Cu	Fe	Mn	Ni	Pb	Zn	
SR1	3.90E-01	2.53E-03	2.43E-04	5.58E-02	1.56E-03	1.12E-01	5.74E-02	1.18E-02	4.70E-01	1.24E-03	1.10
SR2	2.43E-01	1.03E-03	2.62E-04	6.72E-02	1.43E-03	1.09E-01	5.25E-02	1.27E-02	9.44E-02	6.75E-04	0.58
SR3	1.07E+00	6.74E-03	2.02E-04	3.31E-02	2.55E-03	9.79E-02	7.34E-02	7.47E-03	2.04E+00	2.65E-03	3.34
SR4	1.17E-01	2.49E-03	2.19E-04	2.36E-02	1.46E-03	9.81E-02	6.37E-02	5.57E-03	7.07E-02	1.31E-03	0.38
SR5	2.91E-01	9.99E-03	2.55E-04	2.30E-02	1.82E-03	1.14E-01	9.81E-02	7.57E-03	1.45E-01	3.45E-03	0.69
SR6	1.06E+00	3.94E-03	1.76E-04	2.46E-02	2.02E-03	8.43E-02	7.37E-02	4.99E-03	4.19E+00	2.81E-03	5.44
SR7	5.43E-01	2.67E-03	2.10E-04	3.50E-02	2.01E-03	9.87E-02	6.37E-02	7.59E-03	2.09E+00	1.50E-03	2.85
Max	1.07E+00	9.99E-03	2.62E-04	6.72E-02	2.55E-03	1.14E-01	9.81E-02	1.27E-02	4.19E+00	3.45E-03	5.44
Min	1.17E-01	1.03E-03	1.76E-04	2.30E-02	1.43E-03	8.43E-02	5.25E-02	4.99E-03	7.07E-02	6.75E-04	0.38
Mean	5.30E-01	4.20E-03	2.24E-04	3.75E-02	1.84E-03	1.02E-01	6.89E-02	8.24E-03	1.30E+00	1.95E-03	2.06

Table S28. Average daily dose (ADD) for dermal exposure to PTEs

Sampling site	Elements									
	As	Cd	Co	Cr	Cu	Fe	Mn	Ni	Pb	Zn
SR1	1.40E-05	1.01E-08	1.36E-07	6.68E-07	2.49E-07	3.12E-04	6.86E-06	9.40E-07	6.56E-06	1.49E-06
SR2	8.71E-06	4.11E-09	1.46E-07	8.04E-07	2.29E-07	3.05E-04	6.29E-06	1.01E-06	1.32E-06	8.08E-07
SR3	3.84E-05	2.69E-08	1.13E-07	3.96E-07	4.07E-07	2.73E-04	8.78E-06	5.96E-07	2.86E-05	3.18E-06
SR4	4.19E-06	9.95E-09	1.22E-07	2.83E-07	2.32E-07	2.74E-04	7.63E-06	4.44E-07	9.88E-07	1.56E-06
SR5	1.04E-05	3.99E-08	1.42E-07	2.75E-07	2.91E-07	3.17E-04	1.17E-05	6.04E-07	2.03E-06	4.13E-06
SR6	3.80E-05	1.57E-08	9.81E-08	2.94E-07	3.23E-07	2.36E-04	8.82E-06	3.98E-07	5.85E-05	3.37E-06
SR7	1.95E-05	1.06E-08	1.17E-07	4.19E-07	3.21E-07	2.76E-04	7.62E-06	6.06E-07	2.92E-05	1.79E-06
Max	3.84E-05	3.99E-08	1.46E-07	8.04E-07	4.07E-07	3.17E-04	1.17E-05	1.01E-06	5.85E-05	4.13E-06
Min	4.19E-06	4.11E-09	9.81E-08	2.75E-07	2.29E-07	2.36E-04	6.29E-06	3.98E-07	9.88E-07	8.08E-07
Mean	1.90E-05	1.68E-08	1.25E-07	4.49E-07	2.93E-07	2.85E-04	8.25E-06	6.57E-07	1.82E-05	2.33E-06

Table S29. Hazard Quotient (HQ) and Hazards Index for dermal exposure to PTEs

Sampling site	HQ										HI
	As	Cd	Co	Cr	Cu	Fe	Mn	Ni	Pb	Zn	
SR1	4.91E-02	1.26E-04	3.88E-06	1.71E-02	2.08E-05	3.18E-02	5.72E-03	1.17E-03	1.88E-03	8.13E-06	0.11
SR2	3.06E-02	5.14E-05	4.17E-06	2.06E-02	1.90E-05	3.11E-02	5.24E-03	1.27E-03	3.77E-04	4.42E-06	0.09
SR3	1.35E-01	3.36E-04	3.23E-06	1.02E-02	3.39E-05	2.79E-02	7.32E-03	7.45E-04	8.16E-03	1.74E-05	0.19
SR4	1.47E-02	1.24E-04	3.50E-06	7.26E-03	1.94E-05	2.80E-02	6.36E-03	5.55E-04	2.82E-04	8.54E-06	0.06
SR5	3.66E-02	4.98E-04	4.07E-06	7.06E-03	2.42E-05	3.24E-02	9.79E-03	7.55E-04	5.79E-04	2.26E-05	0.09
SR6	1.33E-01	1.97E-04	2.80E-06	7.54E-03	2.69E-05	2.40E-02	7.35E-03	4.98E-04	1.67E-02	1.84E-05	0.19
SR7	6.84E-02	1.33E-04	3.35E-06	1.07E-02	2.68E-05	2.81E-02	6.35E-03	7.58E-04	8.35E-03	9.80E-06	0.12
Max	1.35E-01	4.98E-04	4.17E-06	2.06E-02	3.39E-05	3.24E-02	9.79E-03	1.27E-03	1.67E-02	2.26E-05	0.19
Min	1.47E-02	5.14E-05	2.80E-06	7.06E-03	1.90E-05	2.40E-02	5.24E-03	4.98E-04	2.82E-04	4.42E-06	0.06
Mean	6.68E-02	2.09E-04	3.57E-06	1.15E-02	2.44E-05	2.90E-02	6.88E-03	8.22E-04	5.19E-03	1.27E-05	0.12

Table S30. Average daily dose (ADD) for inhalation exposure to PTEs

Sampling site	Elements									
	As	Cd	Co	Cr	Cu	Fe	Mn	Ni	Pb	Zn
SR1	1.72E-08	3.72E-10	5.01E-09	2.46E-08	9.19E-09	1.15E-05	2.53E-07	3.46E-08	2.42E-07	5.48E-08
SR2	1.07E-08	1.52E-10	5.39E-09	2.96E-08	8.42E-09	1.12E-05	2.32E-07	3.74E-08	4.86E-08	2.98E-08
SR3	4.71E-08	9.91E-10	4.17E-09	1.46E-08	1.50E-08	1.01E-05	3.24E-07	2.20E-08	1.05E-06	1.17E-07
SR4	5.15E-09	3.67E-10	4.51E-09	1.04E-08	8.57E-09	1.01E-05	2.81E-07	1.64E-08	3.64E-08	5.76E-08
SR5	1.28E-08	1.47E-09	5.25E-09	1.02E-08	1.07E-08	1.17E-05	4.33E-07	2.23E-08	7.47E-08	1.52E-07
SR6	4.67E-08	5.80E-10	3.62E-09	1.08E-08	1.19E-08	8.68E-06	3.25E-07	1.47E-08	2.16E-06	1.24E-07
SR7	2.39E-08	3.92E-10	4.32E-09	1.54E-08	1.18E-08	1.02E-05	2.81E-07	2.23E-08	1.08E-06	6.61E-08
Max	4.71E-08	1.47E-09	5.39E-09	2.96E-08	1.50E-08	1.17E-05	4.33E-07	3.74E-08	2.16E-06	1.52E-07
Min	5.15E-09	1.52E-10	3.62E-09	1.02E-08	8.42E-09	8.68E-06	2.32E-07	1.47E-08	3.64E-08	2.98E-08
Mean	2.34E-08	6.17E-10	4.61E-09	1.65E-08	1.08E-08	1.05E-05	3.04E-07	2.42E-08	6.70E-07	8.60E-08

Table S31. Hazard Quotient (HQ) and Hazards Index for inhalation exposure to PTEs

Sampling site	HQ										HI
	As	Cd	Co	Cr	Cu	Fe	Mn	Ni	Pb	Zn	
SR1	1.72E-05	3.72E-07	8.77E-04	8.61E-04	2.29E-07	N/D	1.77E-02	1.68E-06	6.87E-05	1.83E-07	1.95E-02
SR2	1.07E-05	1.52E-07	9.43E-04	1.04E-03	2.10E-07	N/D	1.62E-02	1.81E-06	1.38E-05	9.93E-08	1.82E-02
SR3	4.71E-05	9.91E-07	7.30E-04	5.11E-04	3.73E-07	N/D	2.26E-02	1.07E-06	2.99E-04	3.90E-07	2.42E-02
SR4	5.15E-06	3.67E-07	7.91E-04	3.65E-04	2.13E-07	N/D	1.97E-02	7.95E-07	1.03E-05	1.92E-07	2.08E-02
SR5	1.28E-05	1.47E-06	9.19E-04	3.55E-04	2.67E-07	N/D	3.03E-02	1.08E-06	2.12E-05	5.08E-07	3.16E-02
SR6	4.67E-05	5.80E-07	6.33E-04	3.79E-04	2.96E-07	N/D	2.27E-02	7.13E-07	6.12E-04	4.14E-07	2.44E-02
SR7	2.39E-05	3.92E-07	7.56E-04	5.40E-04	2.95E-07	N/D	1.96E-02	1.08E-06	3.06E-04	2.20E-07	2.13E-02
Max	4.71E-05	1.47E-06	9.43E-04	1.04E-03	3.73E-07	N/D	3.03E-02	1.81E-06	6.12E-04	5.08E-07	3.16E-02
Min	5.15E-06	1.52E-07	6.33E-04	3.55E-04	2.10E-07	N/D	1.62E-02	7.13E-07	1.03E-05	9.93E-08	1.82E-02
Mean	2.34E-05	6.17E-07	8.07E-04	5.78E-04	2.69E-07	N/D	2.13E-02	1.18E-06	1.90E-04	2.87E-07	2.29E-02

N/D – not determinated

Table S32. Estimated carcinogenic risk (CR) for As, Pb, Cd, and Ni via different exposure pathways

Sampling site	As $_{(ing)}$ [a]	As $_{(ing)}$ [b]	As $_{(derm)}$	As $_{(inh)}$	Pb $_{(ing)}$	Pb $_{(derm)}$	Pb $_{(inh)}$	Cd $_{(inh)}$	Ni $_{(inh)}$	CRtot[a]	CRtot[b]
SR1	3.74E-03	1.75E-04	2.10E-05	2.06E-07	1.40E-05	2.76E-07	1.02E-08	2.34E-09	3.15E-08	3.74E-03	1.75E-04
SR2	2.33E-03	1.09E-04	1.31E-05	1.28E-07	2.81E-06	5.54E-08	2.04E-09	9.55E-10	3.40E-08	2.33E-03	1.09E-04
SR3	1.03E-02	4.81E-04	5.76E-05	5.66E-07	6.08E-05	1.20E-06	4.42E-08	6.24E-09	2.00E-08	1.03E-02	4.81E-04
SR4	1.12E-03	5.25E-05	6.29E-06	6.18E-08	2.10E-06	4.15E-08	1.53E-09	2.31E-09	1.49E-08	1.12E-03	5.26E-05
SR5	2.79E-03	1.31E-04	1.57E-05	1.54E-07	4.32E-06	8.51E-08	3.14E-09	9.26E-09	2.02E-08	2.79E-03	1.31E-04
SR6	1.02E-02	4.77E-04	5.71E-05	5.61E-07	1.25E-04	2.46E-06	9.05E-08	3.65E-09	1.34E-08	1.02E-02	4.77E-04
SR7	5.21E-03	2.44E-04	2.92E-05	2.87E-07	6.22E-05	1.23E-06	4.52E-08	2.47E-09	2.03E-08	5.21E-03	2.44E-04
Max	1.03E-02	4.81E-04	5.76E-05	5.66E-07	1.25E-04	2.46E-06	9.05E-08	9.26E-09	3.40E-08	5.21E-03	2.44E-04
Min	1.12E-03	5.25E-05	6.29E-06	6.18E-08	2.10E-06	4.15E-08	1.53E-09	9.55E-10	1.34E-08	3.74E-03	1.75E-04
Mean	5.09E-03	2.39E-04	2.85E-05	2.81E-07	3.87E-05	7.63E-07	2.81E-08	3.89E-09	2.21E-08	4.48E-03	2.10E-04

For As, carcinogenic risk for oral exposure was calculated based on updated [44] (a) and previous [45] (b) slope factor values
